# Machine learning-derived phenotypic trajectories of asthma and allergy in children and adolescents: protocol for a systematic review

**DOI:** 10.1136/bmjopen-2023-080263

**Published:** 2024-08-30

**Authors:** Daniil Lisik, Gregorio Paolo Milani, Michael Salisu, Saliha Selin Özuygur Ermis, Emma Goksör, Rani Basna, Göran Wennergren, Hannu Kankaanranta, Bright I Nwaru

**Affiliations:** 1 Krefting Research Centre, Institute of Medicine, Sahlgrenska Academy, University of Gothenburg, Gothenburg, Sweden; 2 Department of Clinical Science and Community Health, University of Milan, Milan, Italy; 3 Pediatric Unit, Ospedale Maggiore Policlinico, Milano, Italy; 4 Department of Pediatrics, University of Gothenburg Sahlgrenska Academy, Gothenburg, Sweden; 5 Department of Clinical Sciences, Lund University, Lund, Sweden; 6 Tampere University Respiratory Research Group, Faculty of Medicine and Health Technology, Tampere University, Tampere, Finland; 7 Wallenberg Centre for Molecular and Translational Medicine, University of Gothenburg, Gothenburg, Sweden

**Keywords:** Allergy, Asthma, Systematic Review, Risk Factors, Meta-Analysis

## Abstract

**Abstract:**

**Introduction:**

Development of asthma and allergies in childhood/adolescence commonly follows a sequential progression termed the ‘atopic march’. Recent reports indicate, however, that these diseases are composed of multiple distinct phenotypes, with possibly differential trajectories. We aim to synthesise the current literature in the field of machine learning-based trajectory studies of asthma/allergies in children and adolescents, summarising the frequency, characteristics and associated risk factors and outcomes of identified trajectories and indicating potential directions for subsequent research in replicability, pathophysiology, risk stratification and personalised management. Furthermore, methodological approaches and quality will be critically appraised, highlighting trends, limitations and future perspectives.

**Methods and analyses:**

10 databases (CAB Direct, CINAHL, Embase, Google Scholar, PsycInfo, PubMed, Scopus, Web of Science, WHO Global Index Medicus and WorldCat Dissertations and Theses) will be searched for observational studies (including conference abstracts and grey literature) from the last 10 years (2013–2023) without restriction by language. Screening, data extraction and assessment of quality and risk of bias (using a custom-developed tool) will be performed independently in pairs. The characteristics of the derived trajectories will be narratively synthesised, tabulated and visualised in figures. Risk factors and outcomes associated with the trajectories will be summarised and pooled estimates from comparable numerical data produced through random-effects meta-analysis. Methodological approaches will be narratively synthesised and presented in tabulated form and figure to visualise trends.

**Ethics and dissemination:**

Ethical approval is not warranted as no patient-level data will be used. The findings will be published in an international peer-reviewed journal.

**PROSPERO registration number:**

CRD42023441691.

**STRENGTHS AND LIMITATIONS OF THIS STUDY**

- 10 databases, including of grey literature, will be searched using exhaustive queries with no limitation by language to encompass all relevant literature.
- Study quality and risk of bias will be assessed thoroughly through a form based on an in-depth review and compilation of related guidelines, checklists and quality assessment tools.
- Two reviewers will independently perform screening, data extraction and quality assessment, minimising the risk of systematic/non-systematic bias and error.
- The explorative nature and data of the investigated literature will limit comparative analysis of computational methodology and characteristics/frequency of the derived trajectories.

## Introduction

 Asthma and allergic diseases, such as atopic dermatitis, allergic rhinitis and food allergy, are among the most common non-communicable paediatric diseases and constitute a substantial public health burden. Prevalence varies widely across regions, but globally, about 10% report having ever had asthma or eczema by the age of 13–14 years, while around 15% report of ever having had hay fever.^1^ Food allergy, in turn, is reported by roughly 5% of children and adolescents.^2 3^ Often, these diseases develop in a sequential progression, termed the ‘atopic march’, beginning with atopic dermatitis in infancy, followed by food allergy, asthma and allergic rhinitis.^4^,^6^ However, recent studies have highlighted a substantial heterogeneity in the trajectories of allergic diseases, both in terms of composition, sequential order and timing.^5 7^ It has furthermore been suggested that the observed progressions may not in fact be trajectories per se, but rather a manifestation of comorbidities occurring more often in certain individuals at certain ages.^8^ Underlying risk factors have also been demonstrated to be differentially associated with different disease trajectories. For example, breastfeeding has been found to be protective against early transient wheezing, but the association appears to be non-significant for early-persistent and intermediate/late-onset wheezing.^9^

Facilitated by the increase of longitudinal clinical data,^10^ a substantial number of studies characterising trajectories of asthma and allergic diseases have been published, including those using machine learning models.^11^,^18^ The historically dominant hypothesis-driven approach of disease characterisation has commonly been based on the clinical presentation of patients and is susceptible to bias,^19^,^21^ while data-driven approaches, in contrast, have the potential to explore large datasets more effectively and identify novel latent patterns.^22^ Phenotypic trajectories, by capturing dynamics across multiple time points, also enable deeper understanding of disease pathophysiology, optimisation of care, as well as development of prediction models.^10^ Although systematic reviews summarising phenotype discoveries in individual diseases such as asthma (including limited findings on phenotypic trajectories)^21 23^ and risk factors of phenotypic trajectories, for example, wheezing^24^ have been published, the present work will be the first to focus on machine learning-derived phenotypic trajectories in children/adolescents and encompassing a broad spectrum of allergic diseases as well as asthma, thereby providing a comprehensive overview of how these diseases develop during the first 18 years of life.

The primary aim of this systematic review will be to summarise the childhood/adolescence trajectories of asthma and/or allergic disease that have been identified and their characteristics (including with the use of meta-analysis) and frequency. The secondary aim will be to summarise variables and computational approaches used to derive these trajectories, as well as to synthesise the risk factors and outcomes associated with the derived trajectories (including with the use of meta-analysis).

## Methods

This protocol has been outlined in accordance with the Preferred Reporting Items for Systematic Review and Meta-Analysis protocol (PRISMA-P)^25^ guidelines (completed checklist can be found in online supplemental table 1). The final report will be written in accordance with the PRISMA^26^ and the Meta-analysis Of Observational Studies in Epidemiology^27^ reporting guidelines. In addition, the protocol has been prospectively registered in the international prospective register of systematic reviews (PROSPERO).

### Eligibility criteria

The following studies will be considered for inclusion:

- *Study design*: primary longitudinal observational studies in which trajectory-defining data are available from at least two time points in the same subject, with at least 1 year from first to last time point.
- *Population*: children and adolescents (up to 18 years old (ie, trajectory-defining data/follow-up no later than until the age of 18 years)) from population-representative samples. In studies where trajectory-defining data extends beyond the age of 18 years, but there is possibility to extract any useful trajectory characteristics or associated risk factors/outcomes up until the age of 18 years, the study in question will be eligible
- *Objective*: utilisation of machine learning approaches (any data-driven method in which investigated subjects are classified into subgroups/trajectories by an algorithm) to identify and characterise (either through self-report/parental report, clinical assessment/measurement/diagnosis or medical records (from registers)) trajectories (subtyping by temporal data) of asthma (including recurrent episodes of wheezing) and/or allergies (including atopic dermatitis, allergic rhinitis/conjunctivitis/rhinoconjunctivitis, atopic dermatitis and food allergy, as well as (indirect) measurements of allergy, such as allergic sensitisation).

There will be no restriction on sample size. Due to the large and increasing number of studies, particularly in recent years, and the fact that studies commonly employ methods built on previous advancements, we will restrict our searches to studies published in the last 10 years (from 1 January 2013 until the date of respective database search). This will also ensure that the findings reflect recent methodological trends. Studies of any publication status will be considered (relevant articles under embargo will be noted but not assessed further with data extraction, narrative synthesis, quality assessment and the like). Likewise, relevant conference abstracts and abstracts without a full text will be noted but not assessed further. Relevant letters to the editor will be included and synthesised as far as possible as full-length articles. There will be no restriction based on language. Non-English articles will be translated using Google Translate.^28^ Reviews (including systematic reviews) will not be included, but relevant reviews will be screened for relevant literature. Finally, the reference lists of included studies will be screened for additional relevant literature.

### Search strategy and data sources

CAB Direct (including CAB Abstracts and Global Health), CINAHL, Embase, Google Scholar, PubMed, Scopus, Web of Science (including KCI and SciELO) and WHO Global Index Medicus (including AIM (Africa), IMEMR (Eastern Mediterranean), IMSEAR ?(South-East Asia), LILACS (Americas) and WPRIM (Western Pacific)) will be searched using exhaustive queries to capture all relevant literature. Likewise, PsycInfo and WorldCat dissertations and theses will be searched for grey literature. Given the indexing nature of Google Scholar, only the first 300 hits will be retrieved.^29^ The search queries were adapted to the syntax of each database. Likewise, the search queries were modified based on character limit and on the existence/nomenclature of subject headings, filters and the like. The search queries were developed through pilot searches on PubMed in September, 2023 (during which additional relevant keywords were identified and the search queries iteratively refined) and consist of three blocks (‘Asthma and allergies’, ‘Subgrouping and trajectory modelling techniques’ and ‘Age-related inclusion terms’, each comprised of ‘OR’ Boolean operator-separated search terms) concatenated with the ‘AND’ Boolean operator. Where possible and the number of studies exceed 1000 (arbitrary threshold above which substantial benefit is given by limitation of records), a filter was added to exclude adult-only studies. Finally, search results were limited to those published in the last 10 years (from 1 January 2013 until the date of respective database search), where possible through an additional block in the search query. Details of the final search queries are presented in online supplemental table 2A–J.

### De-duplication and screening

Records retrieved from the searches will be imported to EndNote V.21 (Clarivate Analytics, 2023) for semi-automated de-duplication, following a method proposed by Bramer *et al*.^30^ The de-duplicated records will subsequently be screened by pairs of reviewers (DL and GM, DL and MS, and DL and SSÖE) working independently using the Rayyan (https://rayyan.ai) web platform. Screening will be performed in two steps. In the first step, screening will be based on title and abstract, while the second step will consist of full-text assessment. Both steps will be performed in a double-blind fashion, with each reviewer independently evaluating every record for eligibility. Exclusion of records will be done according to the following order: (1) no abstract and no full text; (2) non-original article (ie, duplicate); (3) wrong study design; (4) wrong objective (including the exclusive use of non-machine learning methods, such as by manually defining trajectories) and (5) wrong population.^31^ Following completion, the screening decisions will be unblinded for the other reviewer. Disagreements will be resolved through discussion and arbitration by the principal investigator (PI, BIN), if necessary. In the first step, records that are clearly eligible and records for which there is uncertainty of eligibility will be included to the second step, and cause of exclusion will not be documented. In the second step, records that are eligible will get included in the final manuscript, and each exclusion will be documented and reported (including cause of exclusion) in the supplementary material of the final manuscript (structure shown in online supplemental table 3). A PRISMA flow diagram will be produced to illustrate the screening process in the final manuscript.

### Data extraction

Data extraction will be performed independently in a double-blind fashion by pairs of reviewers (DL and GM and DL and MS), using a Microsoft Excel (Microsoft Corp., 2023) data extraction form (online supplemental file), prospectively piloted and modified by DL, BIN and RB based on relevant articles identified during the PubMed pilot searches. Following completion, the extracted data will be unblinded for the other reviewer. Disagreements will be resolved through discussion and arbitration by the PI (BIN), if necessary. Two attempts will be made to contact the corresponding author in case relevant data are missing.

### Data items

The following data items will be extracted from each included article:

#### General study information

- First author and year of publication.
- Country/countries in which the study was conducted.

#### Subject information

- Number of subjects (included in modelling, at baseline and at end of follow-up, where appropriate).
- Age of subjects (age span in which trajectories were identified).
- Source and characteristics of subjects (eg, if they were derived from a cohort (including cohort abbreviation and link to paper or website with information), if they were selected based on the presence of a condition etc).
- Percentage of recruited subjects that participated in the study at baseline.
- Percentage of drop-outs/withdrawals and summary of discussion regarding potential causes and impact of the missing data.

#### Trajectory-defining data and preprocessing

- Rationale/process for selection of trajectory-defining variables.
- Variables used to define trajectories (including source of data and mechanism of assessment, for example, self-report or clinical assessment).
- Preprocessing performed on such data (eg, imputation, scaling, categorisation, dimensionality reduction, etc, as well as methods for assessing/dealing with time variance, noise/variation in data, etc).
- Reproducibility measures taken (eg, publication of analysis code/data, transparent description of methods or the like).

#### Trajectory modelling

- Rationale/process for selection of trajectory modelling technique(s), including (hyper)parameters.
- Technique(s) (including (hyper)parameters) used.
- Methods for optimising models for the given task/data, avoiding overfitting, etc.
- Methods for selecting optimal technique/number of trajectories.
- Reproducibility measures taken (eg, publication of analysis code/data, transparent description of methods, or the like).

#### Evaluation/validation of trajectories and associated risk factors/outcomes

- External validation (if it was performed, and if so, short description of results).
- Evaluation of clinical, epidemiological or pathophysiological meaning/impact of derived trajectories.
- Associated risk factors investigated (ie, variables investigated as risk factors for subsequently being assigned to the trajectory; including rationale for selection of said variables and methods for assessing association).
- Associated outcomes investigated (ie, variables for which assignment to the trajectory was investigated as a risk factor; as above).
- For each trajectory:
  - The given name(e.g.,‘late-onset eczema’).
  - Percentage of the full study population.
  - Details/timing of characteristics (separated by static (eg, gestational age) and dynamic (eg, frequency of wheezing) characteristics).
  - Point estimate and 95% CI for each investigated risk factor.
  - Point estimate and 95% CI for each investigated outcome.

### Quality assessment

As there is no well-established quality assessment tool specific to studies of (computational) trajectory analysis, and given the specific characteristics of eligible studies, a custom quality assessment tool has been prospectively developed by DL, BIN and RB. The tool is based on the structure and rating system of the Effective Public Health Practice Project (EPHPP)^32^ tool (with some core sections/questions remaining). The sections on methodological aspects of the trajectory exploration ((1) preprocessing; (2) trajectory modelling and (3) evaluation and reporting of results) were based on: related systematic reviews by Bashir *et al*,^33^ Meijs *et al*^34^ and Stafford *et al*^35 36^; a narrative review on computational patient trajectory analyses by Allam *et al*^10^; guidelines for reporting machine learning analyses by Luo *et al*^37^ and Stevens *et al*^38^; quality assessment guidelines for machine learning analyses by Kocak *et al*^39^ and Faes *et al*^40^; and the Guidelines for Reporting on Latent Trajectory Studies checklist by Van de Schoot *et al*.^41^ See online supplemental text for details on the theoretical background and reasoning for each section and item in the quality assessment tool. Each section ((a) selection bias; (b) data collection methods; (c) withdrawals and drop-outs; (d) preprocessing; (e) trajectory modelling; (f) associated risk factors and outcomes and (g) evaluation and reporting of results) will be rated in terms of quality as ‘weak’, ‘moderate’, ‘strong’ or ‘not applicable’. An overall rating will also be given to each study based on the number of ‘weak’ section ratings, following the rating system of the EPHPP tool: ‘weak’ if ≥2 sections, ‘moderate’ if one section and ‘strong’ if no section was rated ‘weak’. We acknowledge that the extensive restructuring of sections and items renders the interpretation of the quality assessment largely different from how the developers of EPHPP intended, including the fact that while the overall rating in the original EPHPP tool is based on six domains, our tool consists of seven domains; thus, statistical possibility of a weaker overall rating is increased.^42^ The quality assessment tool (online supplemental file) was piloted and modified based on relevant articles identified during the PubMed pilot searches. Results of the quality assessment will be presented in a table (structure shown in online supplemental table 4).

Quality and risk of bias in each included study will be assessed independently in a double-blind fashion by the same pairs of reviewers that extracted data from said articles. Following completion, the ratings will be unblinded for the other reviewer. Disagreements will be resolved through discussion and arbitration by the PI (BIN), if necessary.

### Data synthesis and statistical analysis

Extracted data items from each included study will be narratively synthesised and tabulated in a table of characteristics (structure shown in online supplemental table 5), except articles under embargo, conference abstracts and abstracts without a full text, which will only be noted/referenced in the manuscript and in a separate table (structure shown in online supplemental table 6). Line plots will be produced to illustrate: (a) the number of studies published across time; (b) the number of studies using each of the different trajectory modelling techniques across time and (c) the number of studies of low, moderate and high overall quality rating across time. Furthermore, a world map will be drawn, with each country coloured in a shade proportional to the number of studies from said country, to illustrate regional density of conducted research on the topic.

A table (structure shown in online supplemental table 7) will be produced to summarise trajectory-defining characteristics, associated risk factors/outcomes and the frequency at which distinct trajectories have been identified. Depending on the quantity and nature of the findings, additional tables may be produced to summarise, for example, disease-specific trajectories (or combinations thereof). Each section in the table(s) will be populated by one trajectory assessed to be distinct from the other trajectories described across the included studies and in which the ages of the subjects are comparable. The number of studies which have identified said trajectory (based on fraction/composition of identical or similar characteristics, as assessed by DL in agreement with BIN) will be presented. In the middle column, the trajectory characteristics will be described. Dynamic characteristics (eg, frequency of wheezing) will be plotted with one line representing the estimates of each study on the Y-axis (eg, percentage of subjects reporting wheezing) and age on the X-axis, or described narratively, depending on data form/availability. Static characteristics (eg, gestational age) will be presented as the percentage of subjects with said characteristic, together with the corresponding 95% CI, which will be calculated with the Wilson score interval method without continuity correction (suitable in case of small samples or proportions close to 0 or 1, which is expected in the present context).^43 44^ The percentage with 95% CI from individual studies will be separated by a comma. In addition, the pooled percentage with 95% CI will be calculated and presented, where possible (details in paragraph below). In the left column, risk factors (eg, maternal smoking during pregnancy) will be presented with the point estimate and 95% CI from each study separated by a comma, as well as the pooled point estimate and 95% CI, where possible (details in paragraph below). In the right column, outcomes (eg, asthma hospitalisation) will be shown, in a similar fashion as risk factors. The data in the left and right columns will be expressed as risk ratios (RRs) and converted to estimates of RR if needed (details in paragraph below). Characteristics, risk factors and outcomes will be color-coded according to the following domains (based on findings from the PubMed pilot searches as well as domain expertise among the authors; see online supplemental table 8) for more details):

1. Personal data (eg, sex and gestational age).
2. Atopy (eg, assessment through skin prick test).
3. Inflammation (eg, measures of blood neutrophils and eosinophils).
4. Food allergy (including family history, symptoms, diagnosis, healthcare use, medication and (indirect) measure of disease).
5. Atopic dermatitis (including family history, symptoms, diagnosis, healthcare use, medication and (indirect) measure of disease).
6. Allergic rhinitis, conjunctivitis and rhinoconjunctivitis (including family history, symptoms, diagnosis, healthcare use, medication and (indirect) measure of disease).
7. Asthma and wheezing (including family history, symptoms, diagnosis, healthcare use, medication and (indirect) measure of disease).
8. Behavioural and socioeconomic data (eg, absenteeism from school, day-care attendance, etc).
9. Environmental exposure (eg, maternal smoking during pregnancy, exposure to mould at home, diet types and food introduction timing, early childhood infection type/frequency, etc).
10. Comorbidity and related health measures (comorbidities and other health data not directly related to asthma or allergy, for example, body mass index (BMI), height, diabetes, etc).
11. Other (data not fitting elsewhere).

Given the heterogeneous and explorative nature of eligible studies and the aims of the present systematic review, we expect limited possibilities to conduct meta-analysis. Nevertheless, where numerical data on risk factors and outcomes associated with the derived trajectories are deemed comparable (in terms of study population, subject age, trajectory characteristics, control group and risk factor/outcome investigated, as assessed by DL in agreement with BIN), meta-analysis will be performed. Similarly, meta-analysis will be used to pool the percentages of static characteristics in those trajectories for which such data are deemed comparable (in terms of study population, trajectory modelling technique and nature of the specific data, as assessed by DL in agreement with BIN). As the eligible studies are expected to be heterogeneous and estimate varying true effect sizes and percentages, the random-effects model is deemed most appropriate.^45 46^

For the risk factor and outcomes meta-analyses, random-effects robust variance estimation (RVE^47^; *robumeta*^48^ R package) will be used, as it enables the inclusion of statistically dependent effect sizes (eg, based on the same control group, measurements at different time points and related measures of outcome) in the same model,^47^ which is expected to constitute part of the eligible data.^24^ Furthermore, the exact dependence structure does not need to be known when using the RVE method,^49^ and assumptions, such as normal distribution of effect sizes and their estimates, are relaxed.^47^ Pooled point estimates with 95% CI will be produced, using either the ‘CORR’ or ‘HIER’ model weighting scheme, depending on the type of statistical dependency in the included studies, ‘CORR’ being applicable if overall, the meta-analysis data stems from studies that report multiple estimates based on the same subjects, while non-independent data suitable for ‘HIER’ stems from different sets of subjects but share other influences, for example, being evaluated by the same group of researchers and/or using the same protocol/tools.^50^ In case of ‘CORR’ (correlated effects), the default rho value (within-study effect size correlation) of 0.8 will be used.^51^ Separate meta-analyses will be performed for each pair of risk factor/outcome and trajectory, if there are comparable numerical data from ≥2 separate studies.^52 53^ Small sample correction for both the residuals and df, which increases performance in small samples of studies, will be used,^50^ as we expect the number of studies in individual meta-analyses to be relatively low. Heterogeneity will be assessed through calculation of the: (a) proportion of between-study variance not due to random sampling error (I-squared; *I*^2^)^54^; (b) between-study variance (Tau-squared; *τ*^2^).^55^,^57^ Forest plots will be created to present the meta-analysis results using the *forestploter*^58^ R package. The pooled point estimates and corresponding 95% CI will also be displayed in online supplemental table 7. A p value of <0.01 instead of the default threshold of <0.05 will define statistical significance in meta-analyses with Satterthwaite df (*df_Sk_*)<4, as these have been reported to be prone to type I errors.^59 60^ RR will be used as measure of effect due to intuitive interpretation.^61^ Data expressed as incidence rate/risk ratio, prevalence ratio and relative risk ratio will be used without conversion, as these are calculated identically to RR.^62^ Likewise, HR and OR data will be used without conversion as long as the outcome is <15% (at the end of follow-up). In case the outcome is more common (≥15%), estimates of RR will be calculated through the following formulae^63^:

-
$RR\approx \sqrt{OR}$

-
$RR\approx \frac{1-0.5\sqrt{HR}}{1-0.5\sqrt{\frac{1}{HR}}}$

For static characteristics, meta-analysis will be performed using a generalised linear mixed model (GLMM) with logit-transformed percentages from individual studies. GLMM was chosen due to the generally lower risk of bias compared with two-step approaches and suitability for cases where the data contain small sample sizes or high proportions, which is expected in the present work.^64 65^ The Wilson score interval method without continuity correction (suitable in case of small samples or proportions close to 0 or 1, which is expected in the present work)^43 44^ will be used to produce the corresponding 95% CI for the percentage in individual studies. The *meta*^66^ R package will be used for the meta-analyses of static characteristics.

Sensitivity analysis will be performed by repeating each meta-analysis in which ≥2 studies remain after excluding studies given an overall ‘weak’ rating. Publication bias will be assessed in case of ≥10 studies^67^ in individual meta-analyses, using the *metafor*^68^ R package through the means of^69^: (a) visual inspection of asymmetry in funnel plots; (b) statistical tests through Begg and Mazumdar correlation test^70^ and Egger’s regression test.^71^ The trim-and-fill method^72^ will be used to assess how many studies would be needed to normalise an asymmetric funnel plot. The code used to perform the above analyses will be written in R statistical software 4.2.3 (R Core Team, 2023) and together with underlying data made freely available at https://osf.io/ayf35/.

## Discussion

Promising research has been published in the field of trajectory exploration of allergic diseases and asthma, identifying novel and clinically meaningful subgroups. Our work—through the inclusion of a broad set of relevant diseases as well as an exhaustive search in ten databases without restriction by language—will provide a comprehensive overview of the current knowledge and methodological trends on this topic. While a restriction on publication date to the past 10 years will be implemented, the rapidly increasing body of research in this area, together with advancements in trajectory modelling techniques, will ensure a broad coverage of findings with focus on the latest methodological trends, building on previous literature and progress. Given the relative novelty and explorative nature of this area of research, interpretability will be limited due to the lack of well-established methodological principles on which assessment can be made regarding soundness of underlying computational approaches, reproducibility and clinical meaningfulness of the identified trajectories. Furthermore, the quality assessment form developed for the present work itself—although detailed and based on a broad set of guidelines, checklists and reviews—has not been externally validated, which warrants cautious interpretation of the rating results. Finally, as we anticipate low number of studies in most meta-analyses, the reliability of the pooled estimates may be relatively low. While some methods offer more reliable estimation in such scenarios, for example, Bayesian modelling,^73^ the lack of strong priors in this field heavily limits our options. In summary, we believe this systematic review will provide value by summarising the central aspects of recent studies, highlighting repeatedly identified trajectories and their characteristics, as well as outlining methodological trends and limitations and perspectives for future work.

## Ethics and dissemination

Ethical approval is not warranted due to the exclusive use of publicly available aggregated data. The findings in this study will be published in a peer-reviewed journal and underlying data and analysis code made freely available in an online repository (https://osf.io/ayf35/)).

## supplementary material

10.1136/bmjopen-2023-080263 online supplemental file 1
