# Supplementary material for: Machine learning-derived phenotypic trajectories of asthma and allergy in children and adolescents: protocol for a systematic review
Source: BMJ Open. 2024 Aug 30;14(8):e080263. doi: 10.1136/bmjopen-2023-080263 (PMC11367367; doi:10.1136/bmjopen-2023-080263)
Supplement: online supplemental file 1 [file bmjopen-14-8-s001.pdf]

Contents

Supplementary tables 2

Supplementary table 1. PRISMA-P checklist 2

Final search queries 5

Supplementary table 2A. CAB Direct (CAB Abstracts and Global Health) 5

Supplementary table 2B. CINAHL 5

Supplementary table 2C. Embase 7

Supplementary table 2D. Google Scholar 8

Supplementary table 2E. PsycINFO 9

Supplementary table 2F. PubMed 10

Supplementary table 2G. Scopus 11

Supplementary table 2H. Web of Science (Web of Science Core Collection, KCI, and SciELO) 13

Supplementary table 2I. WHO Global Index Medicus 14

Supplementary table 2J. WorldCat Dissertations and Theses 15

Supplementary table 3. Records excluded at full-text assessment (structure) 16

Supplementary table 4. Quality assessment of included studies (structure) 17

Supplementary table 5. Table of characteristics of included studies (structure) 18

Supplementary table 6. Relevant studies excluded from further assessment (structure) 19

Supplementary table 7. Trajectory characteristics and associations (structure) 20

Supplementary table 8. Domains and example variables 21

Supplementary texts 23

Supplementary text 1. Background and reasoning for the structure and rating system in the custom-developed quality assessment form 23

Supplementary tables

Supplementary table 1. PRISMA-P checklist

Section and topic	Item No	Checklist item	Location (section or page/line number)
**ADMINISTRATIVE INFORMATION**			
Title:			
Identification	1a	Identify the report as a protocol of a systematic review	Title
Update	1b	If the protocol is for an update of a previous systematic review, identify as such	Title
Registration	2	If registered, provide the name of the registry (such as PROSPERO) and registration number	Abstract (page 2, line 60); Methods (page 5, lines 143-145)
Authors:			
Contact	3a	Provide name, institutional affiliation, e-mail address of all protocol authors; provide physical mailing address of corresponding author	Title page (page 1, lines 16-20)
Contributions	3b	Describe contributions of protocol authors and identify the guarantor of the review	Author contributions (page 17, lines 524-528)
Amendments	4	If the protocol represents an amendment of a previously completed or published protocol, identify as such and list changes; otherwise, state plan for documenting important protocol amendments	*Not applicable*
Support:			
Sources	5a	Indicate sources of financial or other support for the review	Funding (page 17, lines 531-533)
Sponsor	5b	Provide name for the review funder and/or sponsor	Funding (page 17, line 531-533)
Role of sponsor or funder	5c	Describe roles of funder(s), sponsor(s), and/or institution(s), if any, in developing the protocol	Funding (page 17, lines 533-535)
**INTRODUCTION**			
Rationale	6	Describe the rationale for the review in the context of what is already known	Introduction (page 4, lines 100-102 and 109-115)
Objectives	7	Provide an explicit statement of the question(s) the review will address with reference to participants, interventions, comparators, and outcomes (PICO)	Introduction (page 5, lines 128-133)

METHODS			
Eligibility criteria	8	Specify the study characteristics (such as PICO, study design, setting, time frame) and report characteristics (such as years considered, language, publication status) to be used as criteria for eligibility for the review	Methods > Eligibility criteria (pages 5-6, lines 148-173)
Information sources	9	Describe all intended information sources (such as electronic databases, contact with study authors, trial registers or other grey literature sources) with planned dates of coverage	Methods > Search strategy and data sources (page 6, lines 185-191)
Search strategy	10	Present draft of search strategy to be used for at least one electronic database, including planned limits, such that it could be repeated	Methods > Eligibility criteria (page 6, lines 169-173 and 191); Methods > Search strategy and data sources (pages 6-7, lines 185-206); Supplementary tables 2A-J
Study records:			
Data management	11a	Describe the mechanism(s) that will be used to manage records and data throughout the review	Methods > De-duplication and screening (page 7, lines 209-213); Methods > Data extraction (page 8, lines 234-238)
Selection process	11b	State the process that will be used for selecting studies (such as two independent reviewers) through each phase of the review (that is, screening, eligibility and inclusion in meta-analysis)	Methods > De-duplication and screening (pages 7-8, lines 211-231)
Data collection process	11c	Describe planned method of extracting data from reports (such as piloting forms, done independently, in duplicate), any processes for obtaining and confirming data from investigators	Methods > Data extraction (pages 8, lines 234-241)
Data items	12	List and define all variables for which data will be sought (such as PICO items, funding sources), any pre-planned data assumptions and simplifications	Methods > Data items (pages 8-10, lines 244-292)
Outcomes and prioritization	13	List and define all outcomes for which data will be sought, including prioritization of main and additional outcomes, with rationale	Methods > Data synthesis and statistical analysis (pages 11-13, lines 333-409)
Risk of bias in individual studies	14	Describe anticipated methods for assessing risk of bias of individual studies, including whether this will be done at the outcome or study level, or both; state how this information will be used in data synthesis	Methods > Quality assessment (pages 10-11, lines 295-329)
Data synthesis	15a	Describe criteria under which study data will be quantitatively synthesised	Methods > Data synthesis and statistical analysis (pages 13, lines 397-403)
	15b	If data are appropriate for quantitative synthesis, describe planned summary measures, methods of handling data and methods of combining data from studies, including any planned exploration of consistency (such as I^2, Kendall's τ)	Methods > Data synthesis and statistical analysis (pages 13-15, lines 411-458)

	15c	Describe any proposed additional analyses (such as sensitivity or subgroup analyses, meta-regression)	Methods > Data synthesis and statistical analysis (page 13, lines 403-404; page 15, lines 460-461)
	15d	If quantitative synthesis is not appropriate, describe the type of summary planned	Methods > Data synthesis and statistical analysis (pages 11, lines 333-337)
Meta-bias(es)	16	Specify any planned assessment of meta-bias(es) (such as publication bias across studies, selective reporting within studies)	Methods > Data synthesis and statistical analysis (page 15, lines 461-466)
Confidence in cumulative evidence	17	Describe how the strength of the body of evidence will be assessed (such as GRADE)	*Not applicable*

It is strongly recommended that this checklist be read in conjunction with the PRISMA-P Explanation and Elaboration (cite when available) for important clarification on the items. Amendments to a review protocol should be tracked and dated. The copyright for PRISMA-P (including checklist) is held by the PRISMA-P Group and is distributed under a Creative Commons Attribution Licence 4.0.

From: Shamseer L, Moher D, Clarke M, Ghersi D, Liberati A, Petticrew M, Shekelle P, Stewart L, PRISMA-P Group. Preferred reporting items for systematic review and meta-analysis protocols (PRISMA-P) 2015: elaboration and explanation. BMJ. 2015 Jan 2;349(jan02 1):g7647.

Final search queries

Color/formatting explanation
Green: search block
Purple: subject heading (same as controlled vocabulary and thesaurus)
Gray: free search term (for title an abstract or equivalent)
Red: publication year limitation
Black: parentheses and Boolean operators (**bold**: parentheses and Boolean operators between search blocks)

Supplementary table 2A. CAB Direct (CAB Abstracts and Global Health)

#	Block	Search terms
1	**Asthma and allergies**	**(**asthma OR wheezing OR bronchospasm OR "respiratory sounds" OR "respiratory symptoms" OR eczema OR "atopic dermatitis" OR neurodermatitis OR "allergic rhinitis" OR "hay fever" OR "hay-fever" OR "allergic conjunctivitis" OR "allergic rhinoconjunctivitis" OR "nasal symptoms" OR "ocular symptoms" OR "nasal pruritis" OR "nasal pruritus" OR "nasal congestion" OR "ocular pruritis" OR "ocular pruritus" OR "allergy" OR "atopy" OR "hyperreactivity" OR "hyper-reactivity" OR "hypersensitivity" OR "sensitization" OR "skin prick test"**)**
2	**Subgrouping and trajectory modelling techniques**	**(**"latent class" OR "latent profile" OR "latent transition" OR "latent growth" OR "longitudinal LCA" OR "group-based model" OR "group-based trajectory" OR "growth model" OR "growth mixture" OR "growth curve model" OR "mixture model" OR "finite-mixture model" OR "mixed-effects model" OR "dynamic time warping" OR "time-varying effect model" OR "longitudinal k-means" OR "k-means for longitudinal" OR "discrete wavelet transform" OR "structural equation model" OR "semiparametric model" OR "expectation-maximization" OR "non-negative matrix factorization" OR "Markov model" OR "hierarchical Bayesian" OR "trajectory analysis" OR trajectories OR "cluster analysis" OR phenotypes OR subphenotypes OR subtypes**)**
3	**Age-related inclusion terms**	**(**adolescents OR adolescence OR childhood OR children OR infancy OR infants OR preschool OR pre-school OR school OR early-life OR teens OR teenage OR teenagers OR youth OR puberty OR "birth cohort"**)**
**Full query**		**#1 AND #2 AND #3**<br>(asthma OR wheezing OR bronchospasm OR "respiratory sounds" OR "respiratory symptoms" OR eczema OR "atopic dermatitis" OR neurodermatitis OR "allergic rhinitis" OR "hay fever" OR "hay-fever" OR "allergic conjunctivitis" OR "allergic rhinoconjunctivitis" OR "nasal symptoms" OR "ocular symptoms" OR "nasal pruritis" OR "nasal pruritus" OR "nasal congestion" OR "ocular pruritis" OR "ocular pruritus" OR "allergy" OR "atopy" OR "hyperreactivity" OR "hyper-reactivity" OR "hypersensitivity" OR "sensitization" OR "skin prick test") AND ("latent class" OR "latent profile" OR "latent transition" OR "latent growth" OR "longitudinal LCA" OR "group-based model" OR "group-based trajectory" OR "growth model" OR "growth mixture" OR "growth curve model" OR "mixture model" OR "finite-mixture model" OR "mixed-effects model" OR "dynamic time warping" OR "time-varying effect model" OR "longitudinal k-means" OR "k-means for longitudinal" OR "discrete wavelet transform" OR "structural equation model" OR "semiparametric model" OR "expectation-maximization" OR "non-negative matrix factorization" OR "Markov model" OR "hierarchical Bayesian" OR "trajectory analysis" OR trajectories OR "cluster analysis" OR phenotypes OR subphenotypes OR subtypes) AND (adolescents OR adolescence OR childhood OR children OR infancy OR infants OR preschool OR pre-school OR school OR early-life OR teens OR teenage OR teenagers OR youth OR puberty OR "birth cohort")
**Publication date restriction**		*Select years 2013-2023 in the right-side panel on the search result page (under "Results by Year:")*

Subject headings exist but are searched with using the same syntax as regular search terms.

Supplementary table 2B. CINAHL

#	Block	Search terms
1	**Asthma and allergies**	**(**(MH "Asthma+") OR TI("asthma*" OR "bronchospasm*" OR "bronchial spasm*" OR "bronchoconstriction*" OR "respiratory sound*" OR "respiratory symptom*" OR "airway hyperresponsiveness" OR "airway hyper-responsiveness" OR "respiratory hyperresponsiveness" OR "respiratory hyper-responsiveness" OR "bronchial hyperresponsiveness" OR "bronchial hyper-responsiveness" OR "reactive airway*" OR

		"wheez*") OR AB("asthma*" OR "bronchospasm*" OR "bronchial spasm*" OR "bronchoconstriction*" OR "respiratory sound*" OR "respiratory symptom*" OR "airway hyperresponsiveness" OR "airway hyper-responsiveness" OR "respiratory hyperresponsiveness" OR "respiratory hyper-responsiveness" OR "bronchial hyperresponsiveness" OR "bronchial hyper-responsiveness" OR "reactive airway*" OR "wheez*")<br>OR<br>(MH "Eczema+") OR (MH "Dermatitits, Atopic") OR TI("eczem*" OR "dermatit*" OR "prurigo of Besnier" OR "prurigo Besnier" OR "Besnier's prurigo" OR "neurodermatitis") OR AB("eczem*" OR "dermatit*" OR "prurigo of Besnier" OR "prurigo Besnier" OR "Besnier's prurigo" OR "neurodermatitis")<br>OR<br>(MH "Rhinitis, Allergic, Perennial") OR (MH "Rhinitis, Allergic, Seasonal") OR (MH "Conjunctivitis, Allergic") OR TI("hayfever" OR "hay-fever" OR "rhinitis" OR "conjunctivitis" OR "rhinoconjunctivitis" OR "sinusitis" OR "rhinosinusitis" OR "nasal prurit*" OR "nasal congestion" OR "ocular prurit*" OR "itchy eyes" OR "nasal symptom*" OR "ocular symptom*") OR AB("hayfever" OR "hay-fever" OR "rhinitis" OR "conjunctivitis" OR "rhinoconjunctivitis" OR "sinusitis" OR "rhinosinusitis" OR "nasal prurit*" OR "nasal congestion" OR "ocular prurit*" OR "itchy eyes" OR "nasal symptom*" OR "ocular symptom*")<br>OR<br>(MH "Hypersensitivity+") OR TI("hypersensitivit*" OR "sensitization*" OR "atop*" OR "allerg*" OR "hyperreactivit*" OR "hyper-reactivit*" OR "skin prick test*") OR AB("hypersensitivit*" OR "sensitization*" OR "atop*" OR "allerg*" OR "hyperreactivit*" OR "hyper-reactivit*" OR "skin prick test*")<br>**)**
2	**Subgrouping and trajectory modelling techniques**	**(**TI("trajector*" OR "latent class*" OR "latent profile*" OR "latent transition*" OR "latent growth" OR "longitudinal LCA" OR "LTA" OR "LLCA" OR "LCGA" OR "LCGM" OR "LCGMM" OR "LCMM" OR "LGMM" OR "group-based model*" OR "group-based trajector*" OR "GBTM" OR "growth model*" OR "growth mixture" OR "growth curve model*" OR "GCMM" OR "GMM" OR "mixture model*" OR "finite-mixture model*" OR "mixed-effects model*" OR "dynamic time warp*" OR "DTW" OR "soft-DTW" OR "Fréchet distance*" OR "time-varying effect model*" OR "TVEM" OR "longitudinal k-means" OR "k-means for longitudinal" OR "kml" OR "kmlShape" OR "k-shape" OR "traj" OR "Discrete Wavelet Transform" OR "structural equation model*" OR "semiparametric model*" OR "nonparametric model*" OR "expectation-maximization" OR "non-negative matrix factorization" OR "Markov model*" OR "hierarchical Bayesian") OR AB("trajector*" OR "latent class*" OR "latent profile*" OR "latent transition*" OR "latent growth" OR "longitudinal LCA" OR "LTA" OR "LLCA" OR "LCGA" OR "LCGM" OR "LCGMM" OR "LCMM" OR "LGMM" OR "group-based model*" OR "group-based trajector*" OR "GBTM" OR "growth model*" OR "growth mixture" OR "growth curve model*" OR "GCMM" OR "GMM" OR "mixture model*" OR "finite-mixture model*" OR "mixed-effects model*" OR "dynamic time warp*" OR "DTW" OR "soft-DTW" OR "Fréchet distance*" OR "time-varying effect model*" OR "TVEM" OR "longitudinal k-means" OR "k-means for longitudinal" OR "kml" OR "kmlShape" OR "k-shape" OR "traj" OR "Discrete Wavelet Transform" OR "structural equation model*" OR "semiparametric model*" OR "nonparametric model*" OR "expectation-maximization" OR "non-negative matrix factorization" OR "Markov model*" OR "hierarchical Bayesian")<br>OR<br>(MH "Cluster Analysis+") OR (MH "Phenotype+") OR TI("cluster analys*" OR "phenotype*" OR "subphenotype*" OR "subtype*") OR AB("cluster analys*" OR "phenotype*" OR "subphenotype*" OR "subtype*")**)**
3	**Age-related inclusion terms**	**(**(MH "Child+") OR (MH "Adolescence+") OR (MH "Minors (Legal)") OR TI("childhood" OR "children" OR "infancy" OR "infant*" OR "preschool*" OR "pre-school*" OR "school*" OR "adolescence" OR "adolescent*" OR "early-life" OR "teen*" OR "youth" OR "pubert*" OR "birth cohort") OR AB("childhood" OR "children" OR "infancy" OR "infant*" OR "preschool*" OR "pre-school*" OR "school*" OR "adolescence" OR "adolescent*" OR "early-life" OR "teen*" OR "youth" OR "pubert*" OR "birth cohort")**)**

4	**Exclusion of adult-only studies**	**NOT ((**MH "Adult+") **NOT ((**MH "Child+") OR (MH "Adolescence+") OR (MH "Minors (Legal)")**))**
**Full query**		**(#1 AND #2 AND #3) #4**<br>(((MH "Asthma+") OR TI("asthma*" OR "bronchospasm*" OR "bronchial spasm*" OR "bronchoconstriction*" OR "respiratory sound*" OR "respiratory symptom*" OR "airway hyperresponsiveness" OR "airway hyper-responsiveness" OR "respiratory hyperresponsiveness" OR "respiratory hyper-responsiveness" OR "bronchial hyperresponsiveness" OR "bronchial hyper-responsiveness" OR "reactive airway*" OR "wheez*") OR AB("asthma*" OR "bronchospasm*" OR "bronchial spasm*" OR "bronchoconstriction*" OR "respiratory sound*" OR "respiratory symptom*" OR "airway hyperresponsiveness" OR "airway hyper-responsiveness" OR "respiratory hyperresponsiveness" OR "respiratory hyper-responsiveness" OR "bronchial hyperresponsiveness" OR "bronchial hyper-responsiveness" OR "reactive airway*" OR "wheez*") OR (MH "Eczema+") OR (MH "Dermatitits, Atopic") OR TI("eczem*" OR "dermatit*" OR "prurigo of Besnier" OR "prurigo Besnier" OR "Besnier's prurigo" OR "neurodermatitis") OR AB("eczem*" OR "dermatit*" OR "prurigo of Besnier" OR "prurigo Besnier" OR "Besnier's prurigo" OR "neurodermatitis") OR (MH "Rhinitis, Allergic, Perennial") OR (MH "Rhinitis, Allergic, Seasonal") OR (MH "Conjunctivitis, Allergic") OR TI("hayfever" OR "hay-fever" OR "rhinitis" OR "conjunctivitis" OR "rhinoconjunctivitis" OR "sinusitis" OR "rhinosinusitis" OR "nasal prurit*" OR "nasal congestion" OR "ocular prurit*" OR "itchy eyes" OR "nasal symptom*" OR "ocular symptom*") OR AB("hayfever" OR "hay-fever" OR "rhinitis" OR "conjunctivitis" OR "rhinoconjunctivitis" OR "sinusitis" OR "rhinosinusitis" OR "nasal prurit*" OR "nasal congestion" OR "ocular prurit*" OR "itchy eyes" OR "nasal symptom*" OR "ocular symptom*") OR (MH "Hypersensitivity+") OR TI("hypersensitivit*" OR "sensitization*" OR "atop*" OR "allerg*" OR "hyperreactivit*" OR "hyper-reactivit*" OR "skin prick test*") OR AB("hypersensitivit*" OR "sensitization*" OR "atop*" OR "allerg*" OR "hyperreactivit*" OR "hyper-reactivit*" OR "skin prick test*")) AND (TI("trajector*" OR "latent class*" OR "latent profile*" OR "latent transition*" OR "latent growth" OR "longitudinal LCA" OR "LTA" OR "LLCA" OR "LCGA" OR "LCGM" OR "LCGMM" OR "LCMM" OR "LGMM" OR "group-based model*" OR "group-based trajector*" OR "GBTM" OR "growth model*" OR "growth mixture" OR "growth curve model*" OR "GCMM" OR "GMM" OR "mixture model*" OR "finite-mixture model*" OR "mixed-effects model*" OR "dynamic time warp*" OR "DTW" OR "soft-DTW" OR "Fréchet distance*" OR "time-varying effect model*" OR "TVEM" OR "longitudinal k-means" OR "k-means for longitudinal" OR "kml" OR "kmlShape" OR "k-shape" OR "traj" OR "Discrete Wavelet Transform" OR "structural equation model*" OR "semiparametric model*" OR "nonparametric model*" OR "expectation-maximization" OR "non-negative matrix factorization" OR "Markov model*" OR "hierarchical Bayesian") OR AB("trajector*" OR "latent class*" OR "latent profile*" OR "latent transition*" OR "latent growth" OR "longitudinal LCA" OR "LTA" OR "LLCA" OR "LCGA" OR "LCGM" OR "LCGMM" OR "LCMM" OR "LGMM" OR "group-based model*" OR "group-based trajector*" OR "GBTM" OR "growth model*" OR "growth mixture" OR "growth curve model*" OR "GCMM" OR "GMM" OR "mixture model*" OR "finite-mixture model*" OR "mixed-effects model*" OR "dynamic time warp*" OR "DTW" OR "soft-DTW" OR "Fréchet distance*" OR "time-varying effect model*" OR "TVEM" OR "longitudinal k-means" OR "k-means for longitudinal" OR "kml" OR "kmlShape" OR "k-shape" OR "traj" OR "Discrete Wavelet Transform" OR "structural equation model*" OR "semiparametric model*" OR "nonparametric model*" OR "expectation-maximization" OR "non-negative matrix factorization" OR "Markov model*" OR "hierarchical Bayesian") OR (MH "Cluster Analysis+") OR (MH "Phenotype+") OR TI("cluster analys*" OR "phenotype*" OR "subphenotype*" OR "subtype*") OR AB("cluster analys*" OR "phenotype*" OR "subphenotype*" OR "subtype*")) AND ((MH "Child+") OR (MH "Adolescence+") OR (MH "Minors (Legal)") OR TI("childhood" OR "children" OR "infancy" OR "infant*" OR "preschool*" OR "pre-school*" OR "school*" OR "adolescence" OR "adolescent*" OR "early-life" OR "teen*" OR "youth" OR "pubert*" OR "birth cohort") OR AB("childhood" OR "children" OR "infancy" OR "infant*" OR "preschool*" OR "pre-school*" OR "school*" OR "adolescence" OR "adolescent*" OR "early-life" OR "teen*" OR "youth" OR "pubert*" OR "birth cohort"))) NOT ((MH "Adult+") NOT ((MH "Child+") OR (MH "Adolescence+") OR (MH "Minors (Legal)")))
**Publication date restriction**		*Select years 2013-2023 in the left-side panel on the search result page (under "Limit To")*

Clarification. As CINAHL does not enable searching in titles and abstracts at once, each non-MH search term is repeated twice. **Abbreviations/symbols.** AB: searching in abstracts. MH: searching in CINAHL subject headings. TI: searching in titles.

Supplementary table 2C. Embase

#	Block	Search terms
1	**Asthma and allergies**	**(**'asthma'/exp OR ('asthma*' OR 'bronchospasm*' OR 'bronchial spasm*' OR 'bronchoconstriction*' OR 'respiratory sound*' OR 'respiratory symptom*' OR 'airway hyperresponsiveness' OR 'airway hyper-responsiveness' OR 'respiratory hyperresponsiveness' OR 'respiratory hyper-responsiveness' OR 'bronchial hyperresponsiveness' OR 'bronchial hyper-responsiveness' OR 'reactive airway*' OR 'wheez*'):ti,ab<br>OR<br>'eczema'/exp OR 'atopic dermatitis'/exp OR 'neurodermatitis'/exp OR ('eczem*' OR 'dermatit*' OR 'prurigo of Besnier' OR 'prurigo Besnier' OR 'Besnier* prurigo' OR 'neurodermatitis'):ti,ab<br>OR<br>'rhinitis'/exp OR 'allergic conjunctivitis'/exp OR ('hayfever' OR 'hay-fever' OR 'rhinitis' OR 'conjunctivitis' OR 'rhinoconjunctivitis' OR 'sinusitis' OR 'rhinosinusitis' OR 'nasal prurit*' OR 'nasal congestion' OR 'ocular prurit*' OR 'itchy eyes' OR 'nasal symptom*' OR 'ocular symptom*'):ti,ab<br>OR

		'hypersensitivity'/exp OR ('hypersensitivit*' OR 'sensitization*' OR 'atop*' OR 'allerg*' OR 'hyperreactivit*' OR 'hyper-reactivit*' OR 'skin prick test*'):ti,ab**)**
2	**Subgrouping and trajectory modelling techniques**	**(**('trajector*' OR 'latent class*' OR 'latent profile*' OR 'latent transition*' OR 'latent growth' OR 'longitudinal LCA' OR 'LTA' OR 'LLCA' OR 'LCGA' OR 'LCGM' OR 'LCGMM' OR 'LCMM' OR 'LGMM' OR 'group-based model*' OR 'group-based trajector*' OR 'GBTM' OR 'growth model*' OR 'growth mixture' OR 'growth curve model*' OR 'GCMM' OR 'GMM' OR 'mixture model*' OR 'finite-mixture model*' OR 'mixed-effects model*' OR 'dynamic time warp*' OR 'DTW' OR 'soft-DTW' OR 'Fréchet distance*' OR 'time-varying effect model*' OR 'TVEM' OR 'longitudinal k-means' OR 'k-means for longitudinal' OR 'kml' OR 'kmlShape' OR 'k-shape' OR 'traj' OR 'Discrete Wavelet Transform' OR 'structural equation model*' OR 'semiparametric model*' OR 'nonparametric model*' OR 'expectation-maximization' OR 'non-negative matrix factorization' OR 'Markov model*' OR 'hierarchical Bayesian'):ti,ab<br>OR<br>'cluster analysis'/exp OR 'phenotype'/exp OR ('cluster analys*' OR 'phenotype*' OR 'subphenotype*' OR 'subtype*'):ti,ab**)**
3	**Age-related inclusion terms**	**(**'juvenile'/exp OR ('childhood' OR 'children' OR 'infancy' OR 'infant*' OR 'preschool*' OR 'pre-school*' OR 'school*' OR 'adolescence' OR 'adolescent*' OR 'early-life' OR 'teen*' OR 'youth' OR 'pubert*' OR 'birth cohort'):ti,ab**)**
4	**Exclusion of adult-only studies**	**NOT (**'adult'/exp NOT 'juvenile'/exp**)**
5	**Publication date restriction**	**(**2013:py OR 2014:py OR 2015:py OR 2016:py OR 2017:py OR 2018:py OR 2019:py OR 2020:py OR 2021:py OR 2022:py OR 2023:py**)**
**Full query**		**((#1 AND #2 AND #3) #4) AND #5**<br>('asthma'/exp OR 'asthma*':ti,ab OR 'bronchospasm*':ti,ab OR 'bronchial spasm*':ti,ab OR 'bronchoconstriction*':ti,ab OR 'respiratory sound*':ti,ab OR 'respiratory symptom*':ti,ab OR 'airway hyperresponsiveness':ti,ab OR 'airway hyper-responsiveness':ti,ab OR 'respiratory hyperresponsiveness':ti,ab OR 'respiratory hyper-responsiveness':ti,ab OR 'bronchial hyperresponsiveness':ti,ab OR 'bronchial hyper-responsiveness':ti,ab OR 'reactive airway*':ti,ab OR 'wheez*':ti,ab OR 'eczema'/exp OR 'atopic dermatitis'/exp OR 'neurodermatitis'/exp OR 'eczem*':ti,ab OR 'dermatit*':ti,ab OR 'prurigo of besnier':ti,ab OR 'prurigo besnier':ti,ab OR 'besnier* prurigo':ti,ab OR 'neurodermatitis':ti,ab OR 'rhinitis'/exp OR 'allergic conjunctivitis'/exp OR 'hayfever':ti,ab OR 'hay-fever':ti,ab OR 'rhinitis':ti,ab OR 'conjunctivitis':ti,ab OR 'rhinoconjunctivitis':ti,ab OR 'sinusitis':ti,ab OR 'rhinosinusitis':ti,ab OR 'nasal prurit*':ti,ab OR 'nasal congestion':ti,ab OR 'ocular prurit*':ti,ab OR 'itchy eyes':ti,ab OR 'nasal symptom*':ti,ab OR 'ocular symptom*':ti,ab OR 'hypersensitivity'/exp OR 'hypersensitivit*':ti,ab OR 'sensitization*':ti,ab OR 'atop*':ti,ab OR 'allerg*':ti,ab OR 'hyperreactivit*':ti,ab OR 'hyper-reactivit*':ti,ab OR 'skin prick test*':ti,ab) AND ('trajector*':ti,ab OR 'latent class*':ti,ab OR 'latent profile*':ti,ab OR 'latent transition*':ti,ab OR 'latent growth':ti,ab OR 'longitudinal lca':ti,ab OR 'lta':ti,ab OR 'llca':ti,ab OR 'lcga':ti,ab OR 'lcgm':ti,ab OR 'lcgmm':ti,ab OR 'lcmm':ti,ab OR 'lgmm':ti,ab OR 'group-based model*':ti,ab OR 'group-based trajector*':ti,ab OR 'gbtm':ti,ab OR 'growth model*':ti,ab OR 'growth mixture':ti,ab OR 'growth curve model*':ti,ab OR 'gcmm':ti,ab OR 'gmm':ti,ab OR 'mixture model*':ti,ab OR 'finite-mixture model*':ti,ab OR 'mixed-effects model*':ti,ab OR 'dynamic time warp*':ti,ab OR 'dtw':ti,ab OR 'soft-dtw':ti,ab OR 'fréchet distance*':ti,ab OR 'time-varying effect model*':ti,ab OR 'tvem':ti,ab OR 'longitudinal k-means':ti,ab OR 'k-means for longitudinal':ti,ab OR 'kml':ti,ab OR 'kmlshape':ti,ab OR 'k-shape':ti,ab OR 'traj':ti,ab OR 'discrete wavelet transform':ti,ab OR 'structural equation model*':ti,ab OR 'semiparametric model*':ti,ab OR 'nonparametric model*':ti,ab OR 'expectation-maximization':ti,ab OR 'non-negative matrix factorization':ti,ab OR 'markov model*':ti,ab OR 'hierarchical bayesian':ti,ab OR 'cluster analysis'/exp OR 'phenotype'/exp OR 'cluster analys*':ti,ab OR 'phenotype*':ti,ab OR 'subphenotype*':ti,ab OR 'subtype*':ti,ab) AND ('juvenile'/exp OR 'childhood':ti,ab OR 'children':ti,ab OR 'infancy':ti,ab OR 'infant*':ti,ab OR 'preschool*':ti,ab OR 'pre-school*':ti,ab OR 'school*':ti,ab OR 'adolescence':ti,ab OR 'adolescent*':ti,ab OR 'early-life':ti,ab OR 'teen*':ti,ab OR 'youth':ti,ab OR 'pubert*':ti,ab OR 'birth cohort':ti,ab) NOT ('adult'/exp NOT 'juvenile'/exp) AND (2013:py OR 2014:py OR 2015:py OR 2016:py OR 2017:py OR 2018:py OR 2019:py OR 2020:py OR 2021:py OR 2022:py OR 2023:py)
**Publication date restriction**		*Select years 2013-2023 in the left-side panel on the search result page (under "Publication years")*

/exp: searching in the Emtree and extending search to underlying subject headings. py: restricting search results by publication year. ti,ab: searching in titles and abstracts.

Supplementary table 2D. Google Scholar

<table>
<tr><th>#</th><th>Block</th><th>Search terms</th></tr>
<tr><td>1</td><td>Asthma and allergies</td><td>(asthma|wheezing|eczema|dermatitis|rhinitis|conjunctivitis|allergy|atopy|sensitization)</td></tr>
<tr><td>2</td><td>Subgrouping and trajectory modelling techniques</td><td>(trajectory|"cluster analysis"|subphenotype|phenotype|subtype)</td></tr>
</table>

3	**Age-related inclusion terms**	**(**childhood|child|adolescent|adolescence|infant|teen|preschool|school|early-life|youth|"birth cohort"**)**
**Full query**		**#1 #2 #3**<br>(asthma|wheezing|eczema|dermatitis|rhinitis|conjunctivitis|allergy|atopy|sensitization) (trajectory|"cluster analysis"|subphenotype|phenotype|subtype) (childhood|child|adolescent|adolescence|infant|teen|preschool|school|early-life|youth|"birth cohort")
**Publication date restriction**		*Click "Custom range…" on the left-side panel on the results page and set the range as follows: 2013 in the left field and 2023 in the right field*

Clarification. Note that each space (between the three blocks) is equivalent to the AND Boolean operator. Due to 256 character limitation, the search query has been heavily condensed to the most essential (commonly used and most central to capture) keywords. **Abbreviations/symbols**. |: OR Boolean operator.

Supplementary table 2E. PsycINFO

#	Block	Search terms
1	**Asthma and allergies**	**(**MAINSUBJECT.EXACT("Asthma") OR TI,AB("asthma*") OR TI,AB("bronchospasm*") OR TI,AB("bronchial spasm*") OR TI,AB("bronchoconstriction*") OR TI,AB("respiratory sound*") OR TI,AB("respiratory symptom*") OR TI,AB("airway hyperresponsiveness") OR TI,AB("airway hyper-responsiveness") OR TI,AB("respiratory hyperresponsiveness") OR TI,AB("respiratory hyper-responsiveness") OR TI,AB("bronchial hyperresponsiveness") OR TI,AB("bronchial hyper-responsiveness") OR TI,AB("reactive airway*") OR TI,AB("wheez*")<br>OR<br>MAINSUBJECT.EXACT.EXPLODE("Dermatitis") OR TI,AB("eczem*") OR TI,AB("dermatit*") OR TI,AB("prurigo of Besnier") OR TI,AB("prurigo Besnier") OR TI,AB("Besnier's prurigo") OR TI,AB("neurodermatitis")<br>OR<br>TI,AB("hayfever") OR TI,AB("hay-fever") OR TI,AB("rhinitis") OR TI,AB("conjunctivitis") OR TI,AB("rhinoconjunctivitis") OR TI,AB("sinusitis") OR TI,AB("rhinosinusitis") OR TI,AB("nasal prurit*") OR TI,AB("nasal congestion") OR TI,AB("ocular prurit*") OR TI,AB("itchy eyes") OR TI,AB("nasal symptom*") OR TI,AB("ocular symptom*")<br>OR<br>MAINSUBJECT.EXACT.EXPLODE("Allergic Disorders") OR MAINSUBJECT.EXACT("Anaphylactic Shock") OR TI,AB("hypersensitivit*") OR TI,AB("sensitization*") OR TI,AB("atop*") OR TI,AB("allerg*") OR TI,AB("hyperreactivit*") OR TI,AB("hyper-reactivit*") OR TI,AB("skin prick test*")**)**
2	**Subgrouping and trajectory modelling techniques**	**(**TI,AB("trajector*") OR TI,AB("latent class*") OR TI,AB("latent profile*") OR TI,AB("latent transition*") OR TI,AB("latent growth") OR TI,AB("longitudinal LCA") OR TI,AB("LTA") OR TI,AB("LLCA") OR TI,AB("LCGA") OR TI,AB("LCGM") OR TI,AB("LCGMM") OR TI,AB("LCMM") OR TI,AB("LGMM") OR TI,AB("group-based model*") OR TI,AB("group-based trajector*") OR TI,AB("GBTM") OR TI,AB("growth model*") OR TI,AB("growth mixture") OR TI,AB("growth curve model*") OR TI,AB("GCMM") OR TI,AB("GMM") OR TI,AB("mixture model*") OR TI,AB("finite-mixture model*") OR TI,AB("mixed-effects model*") OR TI,AB("dynamic time warp*") OR TI,AB("DTW") OR TI,AB("soft-DTW") OR TI,AB("Fréchet distance*") OR TI,AB("time-varying effect model*") OR TI,AB("TVEM") OR TI,AB("longitudinal k-means") OR TI,AB("k-means for longitudinal") OR TI,AB("kml") OR TI,AB("kmlShape") OR TI,AB("k-shape") OR TI,AB("traj") OR TI,AB("Discrete Wavelet Transform") OR TI,AB("structural equation model*") OR TI,AB("semiparametric model*") OR TI,AB("nonparametric model*") OR TI,AB("expectation-maximization") OR TI,AB("non-negative matrix factorization") OR TI,AB("Markov model*") OR TI,AB("hierarchical Bayesian")<br>OR<br>MAINSUBJECT.EXACT("Cluster Analysis") OR MAINSUBJECT.EXACT.EXPLODE("Phenotypes") OR TI,AB("cluster analys*") OR TI,AB("phenotype*") OR TI,AB("subphenotype*") OR TI,AB("subtype*")**)**
3	**Age-related inclusion terms**	**(**TI,AB("childhood") OR TI,AB("children") OR TI,AB("infancy") OR TI,AB("infant*") OR TI,AB("preschool*") OR TI,AB("pre-school*") OR TI,AB("school*") OR TI,AB("adolescence") OR TI,AB("adolescent*") OR TI,AB("early-life") OR TI,AB("teen*") OR TI,AB("youth") OR TI,AB("pubert*") OR TI,AB("birth cohort")**)**

**Full query**	**#1 AND #2 AND #3**<br>(MAINSUBJECT.EXACT("Asthma") OR TI,AB("asthma*") OR TI,AB("bronchospasm*") OR TI,AB("bronchial spasm*") OR TI,AB("bronchoconstriction*") OR TI,AB("respiratory sound*") OR TI,AB("respiratory symptom*") OR TI,AB("airway hyperresponsiveness") OR TI,AB("airway hyper-responsiveness") OR TI,AB("respiratory hyperresponsiveness") OR TI,AB("respiratory hyper-responsiveness") OR TI,AB("bronchial hyperresponsiveness") OR TI,AB("bronchial hyper-responsiveness") OR TI,AB("reactive airway*") OR TI,AB("wheez*") OR MAINSUBJECT.EXACT.EXPLODE("Dermatitis") OR TI,AB("eczem*") OR TI,AB("dermatit*") OR TI,AB("prurigo of Besnier") OR TI,AB("prurigo Besnier") OR TI,AB("Besnier's prurigo") OR TI,AB("neurodermatitis") OR TI,AB("hayfever") OR TI,AB("hay-fever") OR TI,AB("rhinitis") OR TI,AB("conjunctivitis") OR TI,AB("rhinoconjunctivitis") OR TI,AB("sinusitis") OR TI,AB("rhinosinusitis") OR TI,AB("nasal prurit*") OR TI,AB("nasal congestion") OR TI,AB("ocular prurit*") OR TI,AB("itchy eyes") OR TI,AB("nasal symptom*") OR TI,AB("ocular symptom*") OR MAINSUBJECT.EXACT.EXPLODE("Allergic Disorders") OR MAINSUBJECT.EXACT("Anaphylactic Shock") OR TI,AB("hypersensitivit*") OR TI,AB("sensitization*") OR TI,AB("atop*") OR TI,AB("allerg*") OR TI,AB("hyperreactivit*") OR TI,AB("hyper-reactivit*") OR TI,AB("skin prick test*")) AND (TI,AB("trajector*") OR TI,AB("latent class*") OR TI,AB("latent profile*") OR TI,AB("latent transition*") OR TI,AB("latent growth") OR TI,AB("longitudinal LCA") OR TI,AB("LTA") OR TI,AB("LLCA") OR TI,AB("LCGA") OR TI,AB("LCGM") OR TI,AB("LCGMM") OR TI,AB("LCMM") OR TI,AB("LGMM") OR TI,AB("group-based model*") OR TI,AB("group-based trajector*") OR TI,AB("GBTM") OR TI,AB("growth model*") OR TI,AB("growth mixture") OR TI,AB("growth curve model*") OR TI,AB("GCMM") OR TI,AB("GMM") OR TI,AB("mixture model*") OR TI,AB("finite-mixture model*") OR TI,AB("mixed-effects model*") OR TI,AB("dynamic time warp*") OR TI,AB("DTW") OR TI,AB("soft-DTW") OR TI,AB("Fréchet distance*") OR TI,AB("time-varying effect model*") OR TI,AB("TVEM") OR TI,AB("longitudinal k-means") OR TI,AB("k-means for longitudinal") OR TI,AB("kml") OR TI,AB("kmlShape") OR TI,AB("k-shape") OR TI,AB("traj") OR TI,AB("Discrete Wavelet Transform") OR TI,AB("structural equation model*") OR TI,AB("semiparametric model*") OR TI,AB("nonparametric model*") OR TI,AB("expectation-maximization") OR TI,AB("non-negative matrix factorization") OR TI,AB("Markov model*") OR TI,AB("hierarchical Bayesian") OR MAINSUBJECT.EXACT("Cluster Analysis") OR MAINSUBJECT.EXACT.EXPLODE("Phenotypes") OR TI,AB("cluster analys*") OR TI,AB("phenotype*") OR TI,AB("subphenotype*") OR TI,AB("subtype*")) AND (TI,AB("childhood") OR TI,AB("children") OR TI,AB("infancy") OR TI,AB("infant*") OR TI,AB("preschool*") OR TI,AB("pre-school*") OR TI,AB("school*") OR TI,AB("adolescence") OR TI,AB("adolescent*") OR TI,AB("early-life") OR TI,AB("teen*") OR TI,AB("youth") OR TI,AB("pubert*") OR TI,AB("birth cohort"))
**Publication date restriction**	*Select years 2013-2023 in the left-side panel on the search result page (under "Publication date")*

Clarification. Exclusion of adult-exclusive studies was not done due to lack of suitable subject headings and low number of results. **Abbreviations/symbols.** MAINSUBJECT.EXACT: searching in subject headings of the APA Thesaurus of Psychological Index Terms - Winter 2023. In instances with a .EXPLODE suffix, the underlying subject headings are also searched. TI,AB: searching in titles and abstracts.

Supplementary table 2F. PubMed

#	Block	Search terms
1	**Asthma and allergies**	**(**Asthma[mh] OR asthma*[tiab] OR bronchospasm*[tiab] OR bronchial spasm*[tiab] OR bronchoconstriction*[tiab] OR respiratory sound*[tiab] OR respiratory symptom*[tiab] OR airway hyperresponsiveness[tiab] OR airway hyper-responsiveness[tiab] OR respiratory hyperresponsiveness[tiab] OR respiratory hyper-responsiveness[tiab] OR bronchial hyperresponsiveness[tiab] OR bronchial hyper-responsiveness[tiab] OR reactive airway*[tiab] OR wheez*[tiab]<br>OR<br>Eczema[mh] OR eczem*[tiab] OR dermatit*[tiab] OR prurigo of Besnier[tiab] OR prurigo Besnier[tiab] OR Besnier's prurigo[tiab] OR neurodermatitis[tiab]<br>OR<br>Rhinitis, Allergic[mh] OR Conjunctivitis, Allergic[mh] OR hayfever[tiab] OR hay-fever[tiab] OR rhinitis[tiab] OR conjunctivitis[tiab] OR rhinoconjunctivitis[tiab] OR sinusitis[tiab] OR rhinosinusitis[tiab] OR nasal prurit*[tiab] OR nasal congestion[tiab] OR ocular prurit*[tiab] OR itchy eyes[tiab] OR nasal symptom*[tiab] OR ocular symptom*[tiab]<br>OR<br>Allergy and Immunology[mh] OR Hypersensitivity[mh] OR hypersensitivit*[tiab] OR sensitization*[tiab] OR atop*[tiab] OR allerg*[tiab] OR hyperreactivit*[tiab] OR hyper-reactivit*[tiab] OR skin prick test*[tiab]**)**
2	**Subgrouping and trajectory modelling techniques**	**(**trajector*[tiab] OR latent class*[tiab] OR latent profile*[tiab] OR latent transition*[tiab] OR latent growth[tiab] OR longitudinal LCA[tiab] OR LTA[tiab] OR LLCA[tiab] OR LCGA[tiab] OR LCGM[tiab] OR LCGMM[tiab] OR LCMM[tiab] OR LGMM[tiab] OR group-based model*[tiab] OR group-based trajector*[tiab] OR GBTM[tiab] OR growth model*[tiab] OR growth mixture[tiab] OR growth curve model*[tiab] OR GCMM[tiab] OR GMM[tiab] OR mixture model*[tiab] OR finite-mixture model*[tiab] OR mixed-effects model*[tiab] OR dynamic time warp*[tiab] OR DTW[tiab] OR soft-DTW[tiab] OR Fréchet distance*[tiab] OR time-varying effect model*[tiab] OR TVEM[tiab] OR longitudinal k-means[tiab] OR k-means for longitudinal[tiab] OR kml[tiab] OR kmlShape[tiab] OR k-shape[tiab] OR traj[tiab] OR Discrete Wavelet Transform[tiab] OR structural equation model*[tiab] OR semiparametric model*[tiab]

		OR nonparametric model*[tiab] OR expectation-maximization[tiab] OR non-negative matrix factorization[tiab] OR Markov model*[tiab] OR hierarchical Bayesian[tiab]<br>OR<br>Cluster Analysis[mh] OR Phenotype[mh] OR cluster analys*[tiab] OR phenotype*[tiab] OR subphenotype*[tiab] OR subtype*[tiab]**)**
3	**Age-related inclusion terms**	**(**Adolescent[mh] OR Child[mh] OR Infant[mh] OR childhood[tiab] OR children[tiab] OR infancy[tiab] OR infant*[tiab] OR preschool*[tiab] OR pre-school*[tiab] OR school*[tiab] OR adolescence[tiab] OR adolescent*[tiab] OR early-life[tiab] OR teen*[tiab] OR youth[tiab] OR pubert*[tiab] OR birth cohort[tiab]**)**
4	**Exclusion of adult-only studies**	**NOT (**Adult[mh] **NOT (**Adolescent[mh] OR Child[mh] OR Infant[mh]**))**
**Full query**		**(#1 AND #2 AND #3) #4**<br>((Asthma[mh] OR asthma*[tiab] OR bronchospasm*[tiab] OR bronchial spasm*[tiab] OR bronchoconstriction*[tiab] OR respiratory sound*[tiab] OR respiratory symptom*[tiab] OR airway hyperresponsiveness[tiab] OR airway hyper-responsiveness[tiab] OR respiratory hyperresponsiveness[tiab] OR respiratory hyper-responsiveness[tiab] OR bronchial hyperresponsiveness[tiab] OR bronchial hyper-responsiveness[tiab] OR reactive airway*[tiab] OR wheez*[tiab] OR Eczema[mh] OR eczem*[tiab] OR dermatit*[tiab] OR prurigo of Besnier[tiab] OR prurigo Besnier[tiab] OR Besnier's prurigo[tiab] OR neurodermatitis[tiab] OR Rhinitis, Allergic[mh] OR Conjunctivitis, Allergic[mh] OR hayfever[tiab] OR hay-fever[tiab] OR rhinitis[tiab] OR conjunctivitis[tiab] OR rhinoconjunctivitis[tiab] OR sinusitis[tiab] OR rhinosinusitis[tiab] OR nasal prurit*[tiab] OR nasal congestion[tiab] OR ocular prurit*[tiab] OR itchy eyes[tiab] OR nasal symptom*[tiab] OR ocular symptom*[tiab] OR Allergy and Immunology[mh] OR Hypersensitivity[mh] OR hypersensitivit*[tiab] OR sensitization*[tiab] OR atop*[tiab] OR allerg*[tiab] OR hyperreactivit*[tiab] OR hyper-reactivit*[tiab] OR skin prick test*[tiab]) AND (trajector*[tiab] OR latent class*[tiab] OR latent profile*[tiab] OR latent transition*[tiab] OR latent growth[tiab] OR longitudinal LCA[tiab] OR LTA[tiab] OR LLCA[tiab] OR LCGA[tiab] OR LCGM[tiab] OR LCGMM[tiab] OR LCMM[tiab] OR LGMM[tiab] OR group-based model*[tiab] OR group-based trajector*[tiab] OR GBTM[tiab] OR growth model*[tiab] OR growth mixture[tiab] OR growth curve model*[tiab] OR GCMM[tiab] OR GMM[tiab] OR mixture model*[tiab] OR finite-mixture model*[tiab] OR mixed-effects model*[tiab] OR dynamic time warp*[tiab] OR DTW[tiab] OR soft-DTW[tiab] OR Fréchet distance*[tiab] OR time-varying effect model*[tiab] OR TVEM[tiab] OR longitudinal k-means[tiab] OR k-means for longitudinal[tiab] OR kml[tiab] OR kmlShape[tiab] OR k-shape[tiab] OR traj[tiab] OR Discrete Wavelet Transform[tiab] OR structural equation model*[tiab] OR semiparametric model*[tiab] OR nonparametric model*[tiab] OR expectation-maximization[tiab] OR non-negative matrix factorization[tiab] OR Markov model*[tiab] OR hierarchical Bayesian[tiab] OR Cluster Analysis[mh] OR Phenotype[mh] OR cluster analys*[tiab] OR phenotype*[tiab] OR subphenotype*[tiab] OR subtype*[tiab]) AND (Adolescent[mh] OR Child[mh] OR Infant[mh] OR childhood[tiab] OR children[tiab] OR infancy[tiab] OR infant*[tiab] OR preschool*[tiab] OR pre-school*[tiab] OR school*[tiab] OR adolescence[tiab] OR adolescent*[tiab] OR early-life[tiab] OR teen*[tiab] OR youth[tiab] OR pubert*[tiab] OR birth cohort[tiab])) NOT (Adult[mh] NOT (Adolescent[mh] OR Child[mh] OR Infant[mh]))
**Publication date restriction**		*Select years 2013-2023 in the left-side panel on the search result page (under "RESULTS BY YEAR")*

mh: searching in Medical Subject Headings (MeSH). tiab: searching in titles and abstracts.

Supplementary table 2G. Scopus

#	Block	Search terms
1	**Asthma and allergies**	**(**TITLE-ABS-KEY("asthma*") OR TITLE-ABS-KEY("bronchospasm*") OR TITLE-ABS-KEY("bronchial spasm*") OR TITLE-ABS-KEY("bronchoconstriction*") OR TITLE-ABS-KEY("respiratory sound*") OR TITLE-ABS-KEY("respiratory symptom*") OR TITLE-ABS-KEY("airway hyperresponsiveness") OR TITLE-ABS-KEY("airway hyper-responsiveness") OR TITLE-ABS-KEY("respiratory hyperresponsiveness") OR TITLE-ABS-KEY("respiratory hyper-responsiveness") OR TITLE-ABS-KEY("bronchial hyperresponsiveness") OR TITLE-ABS-KEY("bronchial hyper-responsiveness") OR TITLE-ABS-KEY("reactive airway*") OR TITLE-ABS-KEY("wheez*")<br>OR<br>TITLE-ABS-KEY("eczem*") OR TITLE-ABS-KEY("dermatit*") OR TITLE-ABS-KEY("prurigo of Besnier") OR TITLE-ABS-KEY("prurigo Besnier") OR TITLE-ABS-KEY("Besnier's prurigo") OR TITLE-ABS-KEY("neurodermatitis")<br>OR<br>TITLE-ABS-KEY("hayfever") OR TITLE-ABS-KEY("hay-fever") OR TITLE-ABS-KEY("rhinitis") OR TITLE-ABS-KEY("conjunctivitis") OR TITLE-ABS-KEY("rhinoconjunctivitis") OR TITLE-ABS-KEY("sinusitis") OR TITLE-ABS-KEY("rhinosinusitis") OR TITLE-ABS-KEY("nasal prurit*") OR TITLE-ABS-KEY("nasal congestion") OR TITLE-ABS-KEY("ocular prurit*") OR TITLE-ABS-KEY("itchy eyes") OR TITLE-ABS-KEY("nasal symptom*") OR TITLE-ABS-KEY("ocular symptom*")

		OR<br>TITLE-ABS-KEY("hypersensitivit*") OR TITLE-ABS-KEY("sensitization*") OR TITLE-ABS-KEY("atop*") OR TITLE-ABS-KEY("allerg*") OR TITLE-ABS-KEY("hyperreactivit*") OR TITLE-ABS-KEY("hyper-reactivit*") OR TITLE-ABS-KEY("skin prick test*")**)**
2	**Subgrouping and trajectory modelling techniques**	**(**TITLE-ABS-KEY("trajector*") OR TITLE-ABS-KEY("latent class*") OR TITLE-ABS-KEY("latent profile*") OR TITLE-ABS-KEY("latent transition*") OR TITLE-ABS-KEY("latent growth") OR TITLE-ABS-KEY("longitudinal LCA") OR TITLE-ABS-KEY("LTA") OR TITLE-ABS-KEY("LLCA") OR TITLE-ABS-KEY("LCGA") OR TITLE-ABS-KEY("LCGM") OR TITLE-ABS-KEY("LCGMM") OR TITLE-ABS-KEY("LCMM") OR TITLE-ABS-KEY("LGMM") OR TITLE-ABS-KEY("group-based model*") OR TITLE-ABS-KEY("group-based trajector*") OR TITLE-ABS-KEY("GBTM") OR TITLE-ABS-KEY("growth model*") OR TITLE-ABS-KEY("growth mixture") OR TITLE-ABS-KEY("growth curve model*") OR TITLE-ABS-KEY("GCMM") OR TITLE-ABS-KEY("GMM") OR TITLE-ABS-KEY("mixture model*") OR TITLE-ABS-KEY("finite-mixture model*") OR TITLE-ABS-KEY("mixed-effects model*") OR TITLE-ABS-KEY("dynamic time warp*") OR TITLE-ABS-KEY("DTW") OR TITLE-ABS-KEY("soft-DTW") OR TITLE-ABS-KEY("Fréchet distance*") OR TITLE-ABS-KEY("time-varying effect model*") OR TITLE-ABS-KEY("TVEM") OR TITLE-ABS-KEY("longitudinal k-means") OR TITLE-ABS-KEY("k-means for longitudinal") OR TITLE-ABS-KEY("kml") OR TITLE-ABS-KEY("kmlShape") OR TITLE-ABS-KEY("k-shape") OR TITLE-ABS-KEY("traj") OR TITLE-ABS-KEY("Discrete Wavelet Transform") OR TITLE-ABS-KEY("structural equation model*") OR TITLE-ABS-KEY("semiparametric model*") OR TITLE-ABS-KEY("nonparametric model*") OR TITLE-ABS-KEY("expectation-maximization") OR TITLE-ABS-KEY("non-negative matrix factorization") OR TITLE-ABS-KEY("Markov model*") OR TITLE-ABS-KEY("hierarchical Bayesian")<br>OR<br>TITLE-ABS-KEY("cluster analys*") OR TITLE-ABS-KEY("phenotype*") OR TITLE-ABS-KEY("subphenotype*") OR TITLE-ABS-KEY("subtype*")**)**
3	**Age-related inclusion terms**	**(**TITLE-ABS-KEY("childhood") OR TITLE-ABS-KEY("children") OR TITLE-ABS-KEY("infancy") OR TITLE-ABS-KEY("infant*") OR TITLE-ABS-KEY("preschool*") OR TITLE-ABS-KEY("pre-school*") OR TITLE-ABS-KEY("school*") OR TITLE-ABS-KEY("adolescence") OR TITLE-ABS-KEY("adolescent*") OR TITLE-ABS-KEY("early-life") OR TITLE-ABS-KEY("teen*") OR TITLE-ABS-KEY("youth") OR TITLE-ABS-KEY("pubert*") OR TITLE-ABS-KEY("birth cohort")**)**
4	**Exclusion of adult-only studies**	**AND NOT (**KEY("adult*") **AND NOT (**KEY("adolescent*") OR KEY("child*") OR KEY("infant*")**))**
5	**Publication date restriction**	**(**PUBYEAR > 2012 AND PUBYEAR < 2024**)**
**Full query**		**((#1 AND #2 AND #3) #4) AND #5**<br>(((TITLE-ABS-KEY ("asthma*") OR TITLE-ABS-KEY ("bronchospasm*") OR TITLE-ABS-KEY ("bronchial spasm*") OR TITLE-ABS-KEY ("bronchoconstriction*") OR TITLE-ABS-KEY ("respiratory sound*") OR TITLE-ABS-KEY ("respiratory symptom*") OR TITLE-ABS-KEY ("airway hyperresponsiveness") OR TITLE-ABS-KEY ("airway hyper-responsiveness") OR TITLE-ABS-KEY ("respiratory hyperresponsiveness") OR TITLE-ABS-KEY ("respiratory hyper-responsiveness") OR TITLE-ABS-KEY ("bronchial hyperresponsiveness") OR TITLE-ABS-KEY ("bronchial hyper-responsiveness") OR TITLE-ABS-KEY ("reactive airway*") OR TITLE-ABS-KEY ("wheez*") OR TITLE-ABS-KEY ("eczem*") OR TITLE-ABS-KEY ("dermatit*") OR TITLE-ABS-KEY ("prurigo of Besnier") OR TITLE-ABS-KEY ("prurigo Besnier") OR TITLE-ABS-KEY ("Besnier's prurigo") OR TITLE-ABS-KEY ("neurodermatitis") OR TITLE-ABS-KEY ("hayfever") OR TITLE-ABS-KEY ("hay-fever") OR TITLE-ABS-KEY ("rhinitis") OR TITLE-ABS-KEY ("conjunctivitis") OR TITLE-ABS-KEY ("rhinoconjunctivitis") OR TITLE-ABS-KEY ("sinusitis") OR TITLE-ABS-KEY ("rhinosinusitis") OR TITLE-ABS-KEY ("nasal prurit*") OR TITLE-ABS-KEY ("nasal congestion") OR TITLE-ABS-KEY ("ocular prurit*") OR TITLE-ABS-KEY ("itchy eyes") OR TITLE-ABS-KEY ("nasal symptom*") OR TITLE-ABS-KEY ("ocular symptom*") OR TITLE-ABS-KEY ("hypersensitivit*") OR TITLE-ABS-KEY ("sensitization*") OR TITLE-ABS-KEY ("atop*") OR TITLE-ABS-KEY ("allerg*") OR TITLE-ABS-KEY ("hyperreactivit*") OR TITLE-ABS-KEY ("hyper-reactivit*") OR TITLE-ABS-KEY ("skin prick test*")) AND (TITLE-ABS-KEY ("trajector*") OR TITLE-ABS-KEY ("latent class*") OR TITLE-ABS-KEY ("latent profile*") OR TITLE-ABS-KEY ("latent transition*") OR TITLE-ABS-KEY ("latent growth") OR TITLE-ABS-KEY ("longitudinal LCA") OR TITLE-ABS-KEY ("LTA") OR TITLE-ABS-KEY ("LLCA") OR TITLE-ABS-KEY ("LCGA") OR TITLE-ABS-KEY ("LCGM") OR TITLE-ABS-KEY ("LCGMM") OR TITLE-ABS-KEY ("LCMM") OR TITLE-ABS-KEY ("LGMM") OR TITLE-ABS-KEY ("group-based model*") OR TITLE-ABS-KEY ("group-based trajector*") OR TITLE-ABS-KEY ("GBTM") OR TITLE-ABS-KEY ("growth model*") OR TITLE-ABS-KEY ("growth mixture") OR TITLE-ABS-KEY ("growth curve model*") OR TITLE-ABS-KEY ("GCMM") OR TITLE-ABS-KEY ("GMM") OR TITLE-ABS-KEY ("mixture model*") OR TITLE-ABS-KEY ("finite-mixture model*") OR TITLE-ABS-KEY ("mixed-effects model*") OR TITLE-ABS-KEY ("dynamic time warp*") OR TITLE-ABS-KEY ("DTW") OR TITLE-ABS-KEY ("soft-DTW") OR TITLE-ABS-KEY ("Fréchet distance*") OR TITLE-ABS-KEY ("time-varying effect model*") OR TITLE-ABS-KEY ("TVEM") OR TITLE-ABS-KEY ("longitudinal k-means") OR TITLE-ABS-KEY ("k-means for longitudinal") OR TITLE-ABS-KEY ("kml") OR TITLE-ABS-KEY ("kmlShape") OR TITLE-ABS-KEY ("k-shape") OR TITLE-ABS-KEY ("traj") OR TITLE-ABS-KEY ("Discrete Wavelet Transform") OR TITLE-ABS-KEY ("structural equation model*") OR TITLE-ABS-KEY ("semiparametric model*") OR TITLE-ABS-KEY ("nonparametric model*") OR TITLE-ABS-KEY ("expectation-maximization") OR TITLE-ABS-KEY ("non-negative matrix factorization") OR TITLE-ABS-KEY ("Markov model*") OR TITLE-ABS-KEY ("hierarchical Bayesian") OR

	TITLE-ABS-KEY ("cluster analys*") OR TITLE-ABS-KEY ("phenotype*") OR TITLE-ABS-KEY ("subphenotype*") OR TITLE-ABS-KEY ("subtype*")) AND (TITLE-ABS-KEY ("childhood") OR TITLE-ABS-KEY ("children") OR TITLE-ABS-KEY ("infancy") OR TITLE-ABS-KEY ("infant*") OR TITLE-ABS-KEY ("preschool*") OR TITLE-ABS-KEY ("pre-school*") OR TITLE-ABS-KEY ("school*") OR TITLE-ABS-KEY ("adolescence") OR TITLE-ABS-KEY ("adolescent*") OR TITLE-ABS-KEY ("early-life") OR TITLE-ABS-KEY ("teen*") OR TITLE-ABS-KEY ("youth") OR TITLE-ABS-KEY ("pubert*") OR TITLE-ABS-KEY ("birth cohort"))) AND NOT (KEY ("adult*") AND NOT (KEY ("adolescent*") OR KEY ("child*") OR KEY ("infant*")))) AND (PUBYEAR > 2012 AND PUBYEAR < 2024)

TITLE-ABS-KEY: searching in title, abstract, and keywords (including controlled vocabulary terms). KEY: searching in keywords (including controlled vocabulary terms). PUBYEAR: limitation of search results by year of publication.

Supplementary table 2H. Web of Science (Web of Science Core Collection, KCI, and SciELO)

In the "DOCUMENTS" tab, click the text to the right of "Search in:" and select "Web of Science Core Collection", "KCI-Korean Journal Database", and "SciELO Citation Index", respectively.

#	Block	Search terms
1	**Asthma and allergies**	**(**TS="asthma*" OR TS="bronchospasm*" OR TS="bronchial spasm*" OR TS="bronchoconstriction*" OR TS="respiratory sound*" OR TS="respiratory symptom*" OR TS="airway hyperresponsiveness" OR TS="airway hyper-responsiveness" OR TS="respiratory hyperresponsiveness" OR TS="respiratory hyper-responsiveness" OR TS="bronchial hyperresponsiveness" OR TS="bronchial hyper-responsiveness" OR TS="reactive airway*" OR TS="wheez*"<br>OR<br>TS="eczem*" OR TS="dermatit*" OR TS="prurigo of Besnier" OR TS="prurigo Besnier" OR TS="Besnier's prurigo" OR TS="neurodermatitis"<br>OR<br>TS="hayfever" OR TS="hay-fever" OR TS="rhinitis" OR TS="conjunctivitis" OR TS="rhinoconjunctivitis" OR TS="sinusitis" OR TS="rhinosinusitis" OR TS="nasal prurit*" OR TS="nasal congestion" OR TS="ocular prurit*" OR TS="itchy eyes" OR TS="nasal symptom*" OR TS="ocular symptom*"<br>OR<br>TS="hypersensitivit*" OR TS="sensitization*" OR TS="atop*" OR TS="allerg*" OR TS="hyperreactivit*" OR TS="hyper-reactivit*" OR TS="skin prick test*"**)**
2	**Subgrouping and trajectory modelling techniques**	**(**TS="trajector*" OR TS="latent class*" OR TS="latent profile*" OR TS="latent transition*" OR TS="latent growth" OR TS="longitudinal LCA" OR TS="LTA" OR TS="LLCA" OR TS="LCGA" OR TS="LCGM" OR TS="LCGMM" OR TS="LCMM" OR TS="LGMM" OR TS="group-based model*" OR TS="group-based trajector*" OR TS="GBTM" OR TS="growth model*" OR TS="growth mixture" OR TS="growth curve model*" OR TS="GCMM" OR TS="GMM" OR TS="mixture model*" OR TS="finite-mixture model*" OR TS="mixed-effects model*" OR TS="dynamic time warp*" OR TS="DTW" OR TS="soft-DTW" OR TS="Fréchet distance*" OR TS="time-varying effect model*" OR TS="TVEM" OR TS="longitudinal k-means" OR TS="k-means for longitudinal" OR TS="kml" OR TS="kmlShape" OR TS="k-shape" OR TS="traj" OR TS="Discrete Wavelet Transform" OR TS="structural equation model*" OR TS="semiparametric model*" OR TS="nonparametric model*" OR TS="expectation-maximization" OR TS="non-negative matrix factorization" OR TS="Markov model*" OR TS="hierarchical Bayesian"<br>OR<br>TS="cluster analys*" OR TS="phenotype*" OR TS="subphenotype*" OR TS="subtype*"**)**
3	**Age-related inclusion terms**	**(**TS="childhood" OR TS="children" OR TS="infancy" OR TS="infant*" OR TS="preschool*" OR TS="pre-school*" OR TS="school*" OR TS="adolescence" OR TS="adolescent*" OR TS="early-life" OR TS="teen*" OR TS="youth" OR TS="pubert*" OR TS="birth cohort"**)**
4	**Exclusion of adult-only studies**	**NOT ((**AK="adult*" OR KP="adult*"**) NOT (**AK="adolescent*" OR KP="adolescent*" OR AK="child*" OR KP="child*" OR AK="infant*" OR KP="infant*"**))**
**Full query**		**(#1 AND #2 AND #3) #4**<br>((TS="asthma*" OR TS="bronchospasm*" OR TS="bronchial spasm*" OR TS="bronchoconstriction*" OR TS="respiratory sound*" OR TS="respiratory symptom*" OR TS="airway hyperresponsiveness" OR TS="airway hyper-responsiveness" OR TS="respiratory hyperresponsiveness" OR TS="respiratory hyper-responsiveness" OR TS="bronchial hyperresponsiveness" OR TS="bronchial hyper-responsiveness" OR TS="reactive airway*" OR

	TS="wheez*" OR TS="eczem*" OR TS="dermatit*" OR TS="prurigo of Besnier" OR TS="prurigo Besnier" OR TS="Besnier's prurigo" OR TS="neurodermatitis" OR TS="hayfever" OR TS="hay-fever" OR TS="rhinitis" OR TS="conjunctivitis" OR TS="rhinoconjunctivitis" OR TS="sinusitis" OR TS="rhinosinusitis" OR TS="nasal prurit*" OR TS="nasal congestion" OR TS="ocular prurit*" OR TS="itchy eyes" OR TS="nasal symptom*" OR TS="ocular symptom*" OR TS="hypersensitivit*" OR TS="sensitization*" OR TS="atop*" OR TS="allerg*" OR TS="hyperreactivit*" OR TS="hyper-reactivit*" OR TS="skin prick test*") AND (TS="trajector*" OR TS="latent class*" OR TS="latent profile*" OR TS="latent transition*" OR TS="latent growth" OR TS="longitudinal LCA" OR TS="LTA" OR TS="LLCA" OR TS="LCGA" OR TS="LCGM" OR TS="LCGMM" OR TS="LCMM" OR TS="LGMM" OR TS="group-based model*" OR TS="group-based trajector*" OR TS="GBTM" OR TS="growth model*" OR TS="growth mixture" OR TS="growth curve model*" OR TS="GCMM" OR TS="GMM" OR TS="mixture model*" OR TS="finite-mixture model*" OR TS="mixed-effects model*" OR TS="dynamic time warp*" OR TS="DTW" OR TS="soft-DTW" OR TS="Fréchet distance*" OR TS="time-varying effect model*" OR TS="TVEM" OR TS="longitudinal k-means" OR TS="k-means for longitudinal" OR TS="kml" OR TS="kmlShape" OR TS="k-shape" OR TS="traj" OR TS="Discrete Wavelet Transform" OR TS="structural equation model*" OR TS="semiparametric model*" OR TS="nonparametric model*" OR TS="expectation-maximization" OR TS="non-negative matrix factorization" OR TS="Markov model*" OR TS="hierarchical Bayesian" OR TS="cluster analys*" OR TS="phenotype*" OR TS="subphenotype*" OR TS="subtype*") AND (TS="childhood" OR TS="children" OR TS="infancy" OR TS="infant*" OR TS="preschool*" OR TS="pre-school*" OR TS="school*" OR TS="adolescence" OR TS="adolescent*" OR TS="early-life" OR TS="teen*" OR TS="youth" OR TS="pubert*" OR TS="birth cohort")) NOT ((AK="adult*" OR KP="adult*") NOT (AK="adolescent*" OR KP="adolescent*" OR AK="child*" OR KP="child*" OR AK="infant*" OR KP="infant*"))
**Publication date restriction**	***Select years 2013 through 2023 under "Publication Years" in the left-side panel on the search result page***

AK: searching in author keywords. KP: searching in Keywords Plus®. TS: searching in title, abstract, author keywords, and Keywords Plus®.

Supplementary table 2I. WHO Global Index Medicus

#	Block	Search terms
1	**Asthma and allergies**	**(**asthma OR wheezing OR bronchospasm OR "respiratory sounds" OR "respiratory symptoms" OR eczema OR "atopic dermatitis" OR neurodermatitis OR "allergic rhinitis" OR "hay fever" OR "hay-fever" OR "allergic conjunctivitis" OR "allergic rhinoconjunctivitis" OR "nasal symptoms" OR "ocular symptoms" OR "nasal pruritis" OR "nasal pruritus" OR "nasal congestion" OR "ocular pruritis" OR "ocular pruritus" OR "allergy" OR "atopy" OR "hyperreactivity" OR "hyper-reactivity" OR "hypersensitivity" OR "sensitization" OR "skin prick test"**)**
2	**Subgrouping and trajectory modelling techniques**	**(**"latent class" OR "latent profile" OR "latent transition" OR "latent growth" OR "longitudinal LCA" OR "group-based model" OR "group-based trajectory" OR "growth model" OR "growth mixture" OR "growth curve model" OR "mixture model" OR "finite-mixture model" OR "mixed-effects model" OR "dynamic time warping" OR "time-varying effect model" OR "longitudinal k-means" OR "k-means for longitudinal" OR "discrete wavelet transform" OR "structural equation model" OR "semiparametric model" OR "expectation-maximization" OR "non-negative matrix factorization" OR "Markov model" OR "hierarchical Bayesian" OR "trajectory analysis" OR trajectories OR "cluster analysis" OR phenotypes OR subphenotypes OR subtypes**)**
3	**Age-related inclusion terms**	**(**adolescents OR adolescence OR childhood OR children OR infancy OR infants OR preschool OR pre-school OR school OR early-life OR teens OR teenage OR teenagers OR youth OR puberty OR "birth cohort"**)**
**Full query**		**#1 AND #2 AND #3**<br>(asthma OR wheezing OR bronchospasm OR "respiratory sounds" OR "respiratory symptoms" OR eczema OR "atopic dermatitis" OR neurodermatitis OR "allergic rhinitis" OR "hay fever" OR "hay-fever" OR "allergic conjunctivitis" OR "allergic rhinoconjunctivitis" OR "nasal symptoms" OR "ocular symptoms" OR "nasal pruritis" OR "nasal pruritus" OR "nasal congestion" OR "ocular pruritis" OR "ocular pruritus" OR "allergy" OR "atopy" OR "hyperreactivity" OR "hyper-reactivity" OR "hypersensitivity" OR "sensitization" OR "skin prick test") AND ("latent class" OR "latent profile" OR "latent transition" OR "latent growth" OR "longitudinal LCA" OR "group-based model" OR "group-based trajectory" OR "growth model" OR "growth mixture" OR "growth curve model" OR "mixture model" OR "finite-mixture model" OR "mixed-effects model" OR "dynamic time warping" OR "time-varying effect model" OR "longitudinal k-means" OR "k-means for longitudinal" OR "discrete wavelet transform" OR "structural equation model" OR "semiparametric model" OR "expectation-maximization" OR "non-negative matrix factorization" OR "Markov model" OR "hierarchical Bayesian" OR "trajectory analysis" OR trajectories OR "cluster analysis" OR phenotypes OR subphenotypes OR subtypes) AND (adolescents OR adolescence OR childhood OR children OR infancy OR infants OR preschool OR pre-school OR school OR early-life OR teens OR teenage OR teenagers OR youth OR puberty OR "birth cohort")
**Publication date restriction**		*Select years 2013-2023 in the left-side panel on the search result page (under "Year range")*

The following fields were searched: title, abstract, and subject descriptor.

Supplementary table 2J. WorldCat Dissertations and Theses

#	Block	Search terms
1	**Asthma and allergies**	**(**asthma OR wheezing OR bronchospasm OR "respiratory sounds" OR "respiratory symptoms" OR eczema OR "atopic dermatitis" OR neurodermatitis OR "allergic rhinitis" OR "hay fever" OR "hay-fever" OR "allergic conjunctivitis" OR "allergic rhinoconjunctivitis" OR "nasal symptoms" OR "ocular symptoms" OR "nasal pruritis" OR "nasal pruritus" OR "nasal congestion" OR "ocular pruritis" OR "ocular pruritus" OR "allergy" OR "atopy" OR "hyperreactivity" OR "hyper-reactivity" OR "hypersensitivity" OR "sensitization" OR "skin prick test"**)**
2	**Subgrouping and trajectory modelling techniques**	**(**"latent class" OR "latent profile" OR "latent transition" OR "latent growth" OR "longitudinal LCA" OR "group-based model" OR "group-based trajectory" OR "growth model" OR "growth mixture" OR "growth curve model" OR "mixture model" OR "finite-mixture model" OR "mixed-effects model" OR "dynamic time warping" OR "time-varying effect model" OR "longitudinal k-means" OR "k-means for longitudinal" OR "discrete wavelet transform" OR "structural equation model" OR "semiparametric model" OR "expectation-maximization" OR "non-negative matrix factorization" OR "Markov model" OR "hierarchical Bayesian" OR "trajectory analysis" OR trajectories OR "cluster analysis" OR phenotypes OR subphenotypes OR subtypes**)**
3	**Age-related inclusion terms**	**(**adolescents OR adolescence OR childhood OR children OR infancy OR infants OR preschool OR pre-school OR school OR early-life OR teens OR teenage OR teenagers OR youth OR puberty OR "birth cohort"**)**
**Full query**		**#1 AND #2 AND #3**<br>(asthma OR wheezing OR bronchospasm OR "respiratory sounds" OR "respiratory symptoms" OR eczema OR "atopic dermatitis" OR neurodermatitis OR "allergic rhinitis" OR "hay fever" OR "hay-fever" OR "allergic conjunctivitis" OR "allergic rhinoconjunctivitis" OR "nasal symptoms" OR "ocular symptoms" OR "nasal pruritis" OR "nasal pruritus" OR "nasal congestion" OR "ocular pruritis" OR "ocular pruritus" OR "allergy" OR "atopy" OR "hyperreactivity" OR "hyper-reactivity" OR "hypersensitivity" OR "sensitization" OR "skin prick test") AND ("latent class" OR "latent profile" OR "latent transition" OR "latent growth" OR "longitudinal LCA" OR "group-based model" OR "group-based trajectory" OR "growth model" OR "growth mixture" OR "growth curve model" OR "mixture model" OR "finite-mixture model" OR "mixed-effects model" OR "dynamic time warping" OR "time-varying effect model" OR "longitudinal k-means" OR "k-means for longitudinal" OR "discrete wavelet transform" OR "structural equation model" OR "semiparametric model" OR "expectation-maximization" OR "non-negative matrix factorization" OR "Markov model" OR "hierarchical Bayesian" OR "trajectory analysis" OR trajectories OR "cluster analysis" OR phenotypes OR subphenotypes OR subtypes) AND (adolescents OR adolescence OR childhood OR children OR infancy OR infants OR preschool OR pre-school OR school OR early-life OR teens OR teenage OR teenagers OR youth OR puberty OR "birth cohort")
**Publication date restriction**		*Select years 2013-2023 in the left-side panel on the search result page (under "Custom Year Range:")*

Supplementary table 3. Records excluded at full-text assessment (structure)

**Record** (first author, year, reference)	**Reason for exclusion**

Supplementary table 4. Quality assessment of included studies (structure)

First author, year, reference	A. Selection bias	B. Data collection methods	C. Withdrawals and drop-outs	D. Preprocessing	E. Trajectory modelling	F. Associated risk factors and outcomes	G. Evaluation and reporting of results	Overall rating

Supplementary table 5. Table of characteristics of included studies (structure)

First author, year, country, reference	Number of subjects[a]	Age of subjects[b]	Source and characteristics of subjects[c]	Input data and preprocessing[d]	Trajectory modelling technique(s)	Definition of optimal technique/number of trajectories	Characteristics of derived trajectories and percentage of full study population[e]	Associated risk factors and outcomes[f]

[a] Denoted as: number of subjects at baseline (number of subjects at end of follow-up, where relevant). [b] Range of age in subjects, denoted as: age (in years, otherwise specified) at first time point of measurement–age (in years, otherwise specified) at last time point of measurement. [c] First, if the subjects were derived from a cohort, the abbreviation of the cohort is given, together with reference to a paper describing the cohort or the official cohort website, if available. Second, general (non-age-related) characteristics of the subjects are described, e.g., if the study population consists of only boys, or if the subjects were derived from a population-based sample based on diagnosis of asthma. [d] First, a general description of the trajectory-defining data is provided, e.g., number of variables and what they intended to measure, assessment method, and frequency of assessment,. Second, a general description of how the data were prepared prior to modelling, e.g., scaling, categorization, dimensionality reduction, and the like. [e] For each of the derived trajectories (named as in the study in question), a general description of the characteristics are provided. First, the dynamic (e.g., frequency of wheezing) characteristics are listed and described generally. Second, the static (e.g., gestational age) characteristics are listed and described generally. Third, the percentage of the trajectory in question of the full study population is stated. [f] For each of the derived trajectories (named as in the study in question), the investigated risk factors and outcomes will be described. First, risk factors (e.g., variables which were investigated as risk factors for being assigned to the trajectory in question) investigated will be listed, together with the point estimate and corresponding 95% confidence interval (95%CI). If a measure of effect other than risk ratio (RR) was used, these data will be recalculated to estimates of RR according to the protocol. Second, outcomes (e.g., variables for which assignment to the trajectory in question was assessed as a risk factor) investigated will be listed, in the same fashion as the risk factors.

Supplementary table 6. Relevant studies excluded from further assessment (structure)

**Record** (first author, year, reference)	**Reason for exclusion**

Supplementary table 7. Trajectory characteristics and associations (structure)

**Name of trajectory** (e.g., "early-onset female-dominant eczema")	**Number of and references to studies that identified said trajectory** (e.g., 4[references])
**Dynamic characteristics** (e.g., frequency of wheezing; if possible, these will be plotted on the y-axis with age on the x-axis, and if not possible, these will be described narratively;iIf multiple studies have found the same dynamic characteristic, these will be plotted togeter, and if not possible, these will be described narratively)<br><br>**List of static characteristics** (e.g., sex; the RR and 95%CI for each study will be presented and separated by a comma; if meta-analysis was performed, the pooled point estimate and 95%CI will be provided as well)<br><br>Personal data<br>**- Female sex**: 90% (56-99),[reference], 88% (79-91),[reference] *pooled 89% (71-93)*[references]<br>**- Prematurity (<37 completed weeks):** 79%[reference]	**List of risk factors** (e.g., prematurity; risk ratio (RR) will be used as measure of effect, with the 95% confidence interval (95%CI) provided and results from multiple studies separated by a comma; if meta-analysis was performed, the pooled point estimate and 95%CI will be provided as well)<br><br>Environmental exposure<br>**- Maternal smoking during pregnancy**: RR 1.98 (1.11–2.67)[reference]<br><br>**List of outcomes** (e.g., asthma diagnosis; risk ratio (RR) will be used as measure of effect, with the 95% confidence interval (95%CI) provided and results from multiple studies separated by a comma; if meta-analysis was performed, the pooled point estimate and 95%CI will be provided as well)<br><br>Asthma<br>**- Physician diagnosis**: RR 1.99 (1.04-2.97)[reference], RR 3.07 (2.55-4.69),[reference] *pooled RR 2.05 (1.42–4.01)*[references]
**Name of next trajectory…**	**…**

95%CI: 95% confidence interval. RR: risk ratio.

Supplementary table 8. Domains and example variables

Domain	Examples of variables
**Personal data**	- Sex<br>- Race<br>- Gestational age<br>- Complications at birth<br>- Genetic variants<br>- Age at puberty/menarche
**Atopy**	- Heredity/affected sibling(s)<br>- Sensitization (skin prick test [SPT], allergen-specific immunoglobulin, E (sIgE) (to specific allergens etc.)<br>- Total immunoglobulin E (tIgE)
**Inflammation**	- Fractional concentration of exhaled nitric oxide (FeNO)<br>- Blood neutrophils, eosinophils etc.<br>- Sputum neutrophils, eosinophils etc.
**Food allergy**	- Heredity/affected sibling(s)<br>- Symptoms (including onset, location, characteristics, intensity, timing, and duration) and (perceived) control of disease<br>- Triggering foods<br>- Diagnosis (including timing and assessment method[s])<br>- Exacerbations and healthcare use (primary care consultation, emergency/acute visit, hospitalization etc., including timing, frequency, and duration)<br>- Medication/treatment (type, dose, duration, tolerance, effect etc.)
**Atopic dermatitis**	- Heredity/affected sibling(s)<br>- Symptoms (including onset, location, characteristics, intensity, timing, and duration) and (perceived) control of disease<br>- Triggering factors<br>- Diagnosis (including timing and assessment method[s])<br>- Exacerbations and health care use (primary care consultation, emergency/acute visit, hospitalization etc., including timing, frequency, and duration)<br>- Medication/treatment (type, dose, duration, tolerance, effect etc.)
**Allergic rhinitis, conjunctivitis, and sinusitis**	- Heredity/affected sibling(s)<br>- Symptoms (including onset, location, characteristics, intensity, timing, and duration) and (perceived) control of disease<br>- Triggering factors<br>- Diagnosis (including timing and assessment method[s])<br>- Exacerbations and health care use (primary care consultation, emergency/acute visit, hospitalization etc., including timing, frequency, and duration)<br>- Medication/treatment (type, dose, duration, tolerance, effect etc.)

**Asthma, wheezing, and lung function**	- Heredity/affected sibling(s)<br>- Symptoms (including onset, location, characteristics, intensity, timing, and duration) and (perceived) control of disease<br>- Lung function measures (forced expiratory volume in the first second [FEV_1], forced vital capacity [FVC], FEV_1/FVC, oscillometry etc), reversibility, hyperreactivity, and the like<br>- Triggering factors<br>- Diagnosis (including timing and assessment method[s])<br>- Exacerbations and health care use (primary care consultation, emergency/acute visit, hospitalization etc., including timing, frequency, and duration)<br>- Medication/treatment (type, dose, duration, tolerance, effect etc.)
**Behavioral and socioeconomic data**	- Child abseentism (from daycare/nursery or school) and parent abseentism (from work)<br>- Psychological status<br>- Daycare attendance<br>- Socioeconomic status
**Environmental exposure**	- Second-hand smoking, maternal smoking during pregnancy, mold, and other air pollution (including duration and intensity of exposure)<br>- Exposure of parents
**Comorbidity and related health measures**	- Diabetes<br>- Height<br>- Obesity/body mass index (BMI) or specific body composition data (e.g., bioimpedance)
**Other**	Variables not fitting elsewhere

Non-exhaustive list of examples of domains and variables. The list of domains and relevant variables may be modified based on findings from eligible papers. Variables in all domains may have any of the following relations with individual trajectories: (a) associated baseline risk-factors, (b) model parameters, or (c) associated outcomes.

Supplementary texts

Supplementary text 1. Background and reasoning for the structure and rating system in the custom-developed quality assessment form

In systematic reviews, the risk of bias (RoB) and the methodological quality in individual studies are commonly assessed using previously established tools. Assessing RoB is essential for interpretation of reported findings as well as for (quantitative) synthesis of results from multiple sources.[1] These tools should be appropriate for the study design(s) of eligible studies[2] and ideally, they should also sufficiently cover the sources of bias identified in quality assessment tools of systematic reviews,[3] such as A MeaSurement Tool to Assess systematic Reviews (AMSTAR) 2 (second version of the AMSTAR tool)[4] and ROBIS.[5] In the context of machine learning-based phenotypic trajectory exploration, however, there is no well-established tool to assess the quality and RoB of such studies, even though a number of reporting guidelines have been published for machine learning analyses in general and for related techniques/contexts,[6-8] as well as a reporting checklist for latent trajectory analyses (Guidelines for Reporting on Latent Trajectory Studies [GRoLTS], by Van de Schoot et al).[9] The paucity of established quality assessment tools for machine learning-based phenotypic trajectory analyses was confirmed in a literature review on PubMed, in which published systematic reviews in closely related contexts ([phenotypic] trajectories and unsupervised prediction models based on longitudinal data) were retrieved. Most existing systematic reviews follow one of two approaches: (1) no RoB of the computational methodology and approaches; (2) RoB with custom-developed versions of well-established quality assessment tools, in which section(s) for assessing such methodological aspects were appended.[10] We opted for the second option, as the interpretation of findings from individual studies is highly dependent on the underlying methods.[11] The GRoLTS checklist was not used as-is to assess quality and RoB because quality of reporting is not equivalent to quality in methodology[2]; rather, the GRoLTS checklist was utilized as reference in defining the machine learning-specific items in the present custom-developed quality assessment tool.

The final quality assessment tool was based on the structure and rating system of the Effective Public Health Practice Project (EPHPP)[12] tool (with some core sections/questions remaining). The sections on methodological aspects of the trajectory analyses were based on: related systematic reviews by Bashir et al.,[13] Meijis et al.,[14] and Stafford et al[15,16]; a narrative review of patient trajectory analyses by Allam et al[17]; guidelines for reporting machine learning analyses by Luo et al.[6] and Stevens et al[7]; quality assessment guidelines for machine learning analyses by Kocak et al.[18] and Faes et al[19]; and the Guidelines for Reporting on Latent Trajectory Studies (GRoLTS) checklist by Van de Schoot et al.[9] Below is described the theoretical background and reasoning for inclusion, definition, and rating system for each item in the custom-developed quality assessment form. Sections/items removed from the original EPHPP tool are marked in ~~red color with strikethrough~~, while added/custom sections/items/definitions are marked in green color. Items are defined in *italic*, with the theoretical background and overall reasoning described below. In the tables, the item and section ratings with definitions are described. In cases where none of the defined "section rating" box combinations of item ratings match the given item ratings, the reviewer is expected to rate the section according to a collected evaluation of the section items ratings and as closely as possible to the defined rating system.

A. Selection bias

A.1. Are the individuals selected to participate in the study likely to be representative of the target population?

For generalizability (external validity) of derived trajectories, it is essential that the study population is representative of the target population.[20]

Option	Value	Definition
1	Very likely	The study population is derived from a comprehensive (or of sufficient size for representatiblity, using random allocation) sampling in the target population. Note that the target population is not necessarily "children in the general population", but can also be e.g., "girls below the age of 18 in the general population", or "asthmatic children in the general population", sampled through a population-based cohort, or the like
2	Somewhat likely	As above, but with some limiations, e.g., the sampling size or certain inclusion/exclusion criteria makes it likely that the study population is not fully representative of the target population
3	Not likely	The study population was selected in a way that substantially negatively impacts its representativeness of the target population

4	Can't tell	Assessment could not be made due to lacking reporting/data

A.2. What percentage of selected individuals agreed to participate?

The percentage of subjects that agree can indicate systematic differences among included and non-included subjects.

Option	Value	Definition
1	80-100%	-
2	60-79%	-
3	<60%	-
4	Not applicable	E.g., in register-based studies in which individual-level agreement was not needed
5	Can't tell	Assessment could not be made due to lacking reporting/data

Section rating

Rating	Definition
Strong	- A.1 is 1 and A.2 is 1, or<br>- A.1 is 1 and A.2 is 4 (in case of e.g,. registry-based studies in which agreement was not relevant on subject-level)
Moderate	- A.1 is 1 or 2 and A.2 is 2, or<br>- A.1 is 2 and A.2 is 1 or 4<br>~~- A.1 is 1 or 2 and A.2 is 5~~ (judged to be too uncertain)
Weak	- A.2 is 3 or 5 (regardless of A.1), or<br>- A.1 is 3 or 4 (regardless of A.2), or<br>- A.1 is 1 and A.2 is 3

~~Study design~~

As this section (in the original EPHPP tool) assesses the likelihood of bias stemming from the allocation process, this section was deemed as irrelevant, given the nature of eligible studies. The study design is of relevance in this context, but primarily through the aspect of data collection (i.e., if the data is prospectively collected, retrieved from registers or healthcare records, or reported by the subjects [risk of recall bias]),[21] which is covered in section B.

~~Confounders~~

This section was substituted with an item assessing management of potential confounders (F.2).

~~Blinding~~

Not relevant in this context as interventional studies are not eligible.

B. Data collection methods

The assessment/measurement/collection methods and sources of data affect validity and reliability, which in turn affect interpretation and strength of findings. Validity is a measure of "appropriateness", more specifically the degree to which measurements measure what the data are supposed to measure.[22]

Reliability, on the other hand, concerns the consitency of data, more specifically to which degree measurements can be replicated in repeated measurements.[23] Importantly, validity implies reliability.[24-26]

B.1. Were methods/tools used to collect trajectory data (and risk factor/outcome data, where relevant) valid?*

* Risk factors are variables for which the association with subsequently being assigned to any of the derived trajectories was investigated (e.g., "premature birth was associated with the transient wheezing trajectory"). Outcomes are variables for which assignment to any of the derived trajectories was investigated as a "risk factor" (e.g., "the transient wheezing trajectory was associated with development of atopic dermatitis at the end of follow-up").

Option	Value	Definition
1	Yes	The data are considered to be valid (and reliable), by clearly assessing the intended trajectory characteristics or investigated risk factors or outcomes
2	No	The data are considered to not be valid, by not (clearly) assessing the intended trajectory characteristics or investigated risk factors or outcomes (e.g., unrelated variables, composite variables that contain substantial noise, or systematically skewed, biased, or unrelated data etc). This option should also be chosen if the data are not relaible
3	Can't tell	Assessment could not be made due to lacking reporting/data

B.2. Were methods/tools used to collect trajectory data (and associated/outcome data, where relevant) reliable?*

* See B.1.

Option	Value	Definition
1	Yes	The data are considered to be reliable. Register-based healthcare data (including relevant diagnosis and procedure codes) as well as clinical assessments of healthcare professionals are examples, as well as, to some degree, self-reported data (in case of clear [preferrably validated] reports derived from questionnaires or interviews regarding events and experiences in present time or near past)
2	No	The data are considered to not be reliable. This primarily concerns self-reported data regarding events and experiences in distant past
3	Can't tell	Assessment could not be made due to lacking reporting/data

Section rating

Rating	Definition
Strong	- B.1 is 1 and B.2 is 1
Moderate	- B.1 is 2 or 3 and B.2 is 1
Weak	- B.2 is 2 or 3 (regardless of B.1)

C. Withdrawals and drop-outs

Trajectory analyses generally become more stable with increased number of time points of measurement.[27] For this reason, it is important to investigate the extent and potential causes of withdrawals/drop-outs, as well as assessing the impact of loss to follow-up, e.g., with sensitivity analysis.

~~*Were withdrawals and drop-outs reported in terms of numbers and/or reasons per group?*~~

Redundant. First and second item in this section (in the original EPHPP tool) were combined into C.1.

~~*Indicate the percentage of participants completing the study. (If the percentage differs by groups, record the lowest).*~~

Redundant. First and second item in this section (in the original EPHPP tool) were combined into C.1.

C.1. What was the percentage of participants completing the study, and were the reasons for withdrawals/drop-outs discussed? (If the percentage of participants completing the study differs by groups, record the lowest.)

**Option**	**Value**	**Definition**
1	80-100%	-
2	60-79%	-
3	<60%	-
4	Not applicable	E.g., in cross-sectional studies
5	Can't tell	Assessment could not be made due to lacking reporting/data (e.g., by absense of discussion regarding reasons for withdrawals/drop-outs)

C.2. Was the impact of loss to follow-up assessed/accounted for, e.g., by investigating differences between subjects lost to follow-up and those who completed the study and with sensitivity analysis?

**Option**	**Value**	**Definition**
1	Yes	Assessment made and appropriately accounted for (if necessary)
2	No	No report or inappropriate/insufficient assessment and/or adjustment in analyses
3	Not applicable	E.g., in cross-sectional studies
4	Can't tell	Assessment could not be made due to lacking reporting/data

Section rating

**Rating**	**Definition**
Strong	- C.1 is 1 or 2 and C.2 is 1
Moderate	- C.1 is 3 and C.1 is 1, or<br>- C.1 is 1 and C.2 is 2 or 4
Weak	- C.1 is 5 (regardless of C.2), or<br>- C.1 is 2 or 3 and C.2 is 2 or 4
Not applicable	- C.1 is 4 or C.2 is 3

D. Preprocessing

D.1. Were missing data assessed, reported, and dealt with appropriately?

Missing data is common in longitudinal settings.[28] The likely missing data mechanism(s) and extent of missingness should be reported. Furthermore, appropriate measures should be taken, where relevant, to account for the missingness. Some trajectory modelling techniques assume certain causes of missingness, such as missing at random (MAR) in latent class growth analysis (LCGA) and latent growth mixture models (LGMM).[9] Thus, the impact of missingness and its underlying (suspected) mechanism(s)

on trajectory modelling technique selection and, where relevant, the impact of the technique(s) utilized to adjust for the missingness on the derived findings ought to be discussed.[7]

Option	Value	Definition
1	Yes	-
2	No	-
3	Can't tell	Assessment could not be made due to lacking reporting/data

D.2. Were the metric of time and time variance (in case of time-unstructured data) reported/handled appropriately?

The spacing in time between measurements can affect the number of trajectories identified. Furthermore, most longitudinal data are time-unstructured (i.e., subjects were not assessed at the same time points), which can cause misinterpretation of derived trajectories.[9]

Option	Value	Definition
1	Yes	One example of suitable handling of metric of time is to specify the exact amount of time passed between each wave; regarding time-unstructured data, e.g., by including a timestamp for each assessment. Also note that this option should be chosen if the spacing between measurements are identical (i.e., 0, 1, 2, 3 etc. in duration) *and* if all subjects were assessed at the same time
2	No	-
3	Can't tell	Assessment could not be made due to lacking reporting/data

D.3. Was noise/variation in data assessed, reported, and dealt with appropriately?

Noise and variation in data should be assessed, e.g., by plotting or tabulating relevant data. Depending on the (suspected) cause(s) of e.g., skewed data, preprocessing may be warranted prior to conducting trajectory analyses, including feature engineering or omitting certain variables.

Option	Value	Definition
1	Yes	-
2	No	-
3	Can't tell	Assessment could not be made due to lacking reporting/data

D.4. Was a statistical, literature, or domain expertise-based approach used in the selection of the trajectory-defining variable(s)?

The selection of trajectory-defining variable(s) should be based on a hypothesis and: (a) a statistical approach (e.g., by including a [complete] set of variables initially and following assessment of impact on results removing variables which do not influence or only marginally influence the model,[6] correlation testing between variables,[7] and the like); on a literature review[29] (identifying previously investigated/established variables of interest); or on domain-expertise (judged by experts in the field to be relevant for testing the present hypothesis).[7]

Option	Value	Definition
1	Yes	-
2	No	-

3	Can't tell	Assessment could not be made due to lacking reporting/data

D.5. Were model input data appropriately prepared, e.g., through dimensionality reduction, coding, scaling, and categorization?

Depending on the nature of data and the trajectory modelling technique utilized,[18] certain variables may necessitate various forms of preprocessing, including scaling, (re-)coding, categorization, and the like. For some techniques, the dimensionality may also be an issue, why dimensionality reduction techniques may be of value, e.g., to avoid "the curse of dimensionality".[30] Dimensionality reduction is also an integral part of certain trajectory modelling techniques and phenotyping approaches.[31] Data of different temporality (e.g., static features vs dynamic [time-dependent] features) may both be used as input, in which case appropriate processing, e.g., with a fusion layer, may be warranted.[17] The central aspect here is that all relevant data transformation should be justified and reported transparently and sufficiently, including details on how the transformation was practically performed.

Option	Value	Definition
1	Yes	-
2	No	-
3	Can't tell	Assessment could not be made due to lacking reporting/data

D.6. Did the author(s) provide sufficient details and/or code to reproduce the steps described in this section?

Providing sufficient details and/or code[32] for the reader to understand and reproduce[33] the undertaken steps is essential in research in general[9,18] and in AI-based analyses specifically.[17] This enables critical appraisal of individual studies, tests of reproducibility, as well as meta-analysis or other forms of (quantitative) synthesis, ultimately accelerating advances in the field.[34]

Option	Value	Definition
1	Yes	Sufficient details or code/container to reproduce the undertaken steps, including the name and version of packages/libraries/software, programming language, as well as the environment/hardware
2	No	-
3	Can't tell	Assessment could not be made due to lacking reporting/data

Section rating

Rating	Definition
Strong	- All items are 1
Moderate	- 1-2 items are 2 or 3 except D.6 (which is 1)
Weak	- ≥3 items are 2 or 3<br>or<br>- D.6 is 2 or 3

E. Trajectory modelling

E.1. Was a literature or domain expertise-based approach used in the selection of trajectory modelling technique(s), including (hyper)parameters?

The choice of trajectory modelling technique(s) heavily impacts what trajectories are derived, as different techniques are based on different underlying mathematical operations, have specific assumptions on input data etc.[17,35] An informed justification, based on previous papers of similar nature (identified through a literature review) and/or domain expertise should constitute the basis for these decisions and ought to be reported.

Option	Value	Definition
1	Yes	-
2	No	-
3	Can't tell	Assessment could not be made due to lacking reporting/data

E.2. Were the methods utilized for assessment and optimization of trajectory modelling technique performance (selection/testing of [hyper]parameters, minimizing risk of overfitting, internal validation, and the like) appropriate?

Depending on the input data and trajectory modelling technique, settings/(hyper)parameters may need tuning to perform the modelling optimally. Furthermore, where relevant, sufficient steps should be taken to avoid overfitting,[16] assess internal validation[7] etc. The methods utilized and the retrieved results should be reported.

Option	Value	Definition
1	Yes	-
2	No	-
3	Can't tell	Assessment could not be made due to lacking reporting/data

E.3. Was the definition of the optimal solution (number of trajectories and/or best-performing trajectory modelling technique) appropriate?

Well-established criterion/criteria, e.g., Bayesian information criterion (BIC), should be used to define the optimal trajectory solution.[9] "Subjective" measures. such as clinical interpretation of trajectories, may also be used, but ideally in combination with appropriate statistical methods.

Option	Value	Definition
1	Yes	-
2	No	-
3	Can't tell	Assessment could not be made due to lacking reporting/data

E.4. Were multiple trajectory modelling techniques used to assess modelling robustness, or a reasonable explanation for abstaining provided?

Although adding substantial weight to an article, assessment of multiple trajectory modelling techniques may be useful to assess if similar trajectories are found using multiple techniques or which technique appears to be most suitable (in unclear cases).

Option	Value	Definition
1	Yes	-
2	No	-

3	Can't tell	Assessment could not be made due to lacking reporting/data

E.5. Did the author(s) provide sufficient details and/or code to reproduce the steps described in this section?

Providing sufficient details and/or code[32] for the reader to understand and reproduce[33] the undertaken steps is essential in research in general[9,18] and in AI-based analyses specifically.[17] This enables critical appraisal of individual studies, tests of reproducibility, as well as meta-analysis or other forms of (quantitative) synthesis, ultimately accelerating advances in the field.[34]

Option	Value	Definition
1	Yes	-
2	No	-
3	Can't tell	Assessment could not be made due to lacking reporting/data

Section rating

Rating	Definition
Strong	- All items are 1 (E.4 can be 2 if using a single technique is justified)
Moderate	- E.1 is 2 or 3 and/or E.4 is 2 or 3, and the remaining items are 1
Weak	- E.2, E.3, or E.5 is 2 or 3 (regardless of the other items)

F. Associated risk factors and outcomes

F.1. Was a literature or domain expertise-based approach used for the selection of investigated risk factors or outcomes?

The basis of testing the association between derived trajectories and risk factors and/or outcomes (see definition in B.1.) should be founded in a hypothesis, further backed by a literature review indiciating a potential association or domain-expertise suggesting a potential association. As observational studies are generally not required to prospectively register protocols or pre-define investigated measures, there is a risk of "recalibration" of research questions and post hoc inclusion of associations that are found during the analysis process to be statistically significant.[36] No silver bullet solution exists to control for such bias, but an informed justification nevertheless brings scientific qualification to the tests performed.

Option	Value	Definition
1	Yes	-
2	No	-
3	Not applicable	Choose this option if no such analyses were performed
4	Can't tell	Assessment could not be made due to lacking reporting/data

F.2. Was the method of assessing the association with the investigated risk factors or outcomes appropriate (e.g., through adjustment of confounders, where relevant)?

In studies where the trajectories are investigated for association with risk factors and/or outcomes (see definition in B.1.), it is essential that a proper statistical method is utilized to derive the estimates of

association, given potential sources of bias, the nature of trajectory data, trajectory modelling technique (including settings/[hyper]parameters), as well as the risk factor(s)/outcome(s) in question.

Option	Value	Definition
1	Yes	-
2	No	-
3	Not applicable	Choose this option if no such analyses were performed
4	Can't tell	Assessment could not be made due to lacking reporting/data

F.3. Was an appropriate approach taken to handle multiplicity bias, where relevant?

In studies where multiple related[37] associations between trajectories and factors or outcomes (see definition in B.1.) are tested, it may be suitable to correct for multiplicity in hypothesis testing,[38] e.g., by using the Bonferroni method.[39]

Option	Value	Definition
1	Yes	-
2	No	-
3	Not applicable	Choose this option if no such analyses were performed
4	Can't tell	Assessment could not be made due to lacking reporting/data

F.4. Did the author(s) provide sufficient details and/or code to reproduce the steps described in this section?

Providing sufficient details and/or code[32] for the reader to understand and reproduce[33] the undertaken steps is essential in research in general[9,18] and in AI-based analyses specifically.[17] This enables critical appraisal of individual studies, tests of reproducibility, as well as meta-analysis or other forms of (quantitative) synthesis, ultimately accelerating advances in the field.[34]

Option	Value	Definition
1	Yes	-
2	No	-
3	Not applicable	Choose this option if no such analyses were performed
4	Can't tell	Assessment could not be made due to lacking reporting/data

Section rating

Rating	Definition
Strong	- All items are 1
Moderate	- F.1 and/or F.3 is 2 or 4 and the remaining items are 1, or<br>- F.1 is 2 or 4 and F.2 and F.4 is 1 (regardless of F.3)
Weak	- F.2 or F.4 is 2 or 4 (regardless of the remaining items)
Not applicable	- All items are 3

G. Evaluation and reporting of results

G.1. Was an evaluation performed of the pathophysiological, clinical, or epidemiological significance of the derived trajectories?

Evaluating the pathophysiological, clinical, or epidemiological significance and meaning of the derived trajectories offers a form of external validation, if indicating that external factors not defining the trajectories in the utilized modelling techniques are significantly and meaningfully associated with the derived trajectories. The scope of this goes beyond mere statistical tests of significance, but rather necessitates a domain-expert assessment of the actual impact of the association(s).

Option	Value	Definition
1	Yes	-
2	No	-
3	Can't tell	Assessment could not be made due to lacking reporting/data

G.2. Was the reporting of results transparent and sufficient, including trajectory plots, characteristics of subjects in the final trajectories, within-class heterogeneity, and the like?

The resulting trajectory data should be clearly reported, including individual trajectories, plots of different trajectory modelling techniques if multiple techniques were assessed/compared, table(s) of characteristics of derived trajectories, and the like. It is essential that potential insufficiencies in the derived trajectories are presented, such as within-class heterogeneity, to facilitate a critical appraisal and interpretation.

Option	Value	Definition
1	Yes	-
2	No	-
3	Can't tell	Assessment could not be made due to lacking reporting/data

G.3. Was an external validation of trajectories performed?

Whenever possible, external validation should be performed,[6] as it is an important generalizability measure of the derived trajectories.[14,19]

Option	Value	Definition
1	Yes	Should be performed on an different dataset/population
2	No	-
3	Can't tell	Assessment could not be made due to lacking reporting/data

G.4. Were the methodological limitations reported?

Transparently providing details on methodological limitations (and deviations from the study protocol, where relevant), whether from limited computational resources, data characteristics, or time frame, provides important insight explaining the nature of the findings, as well as potential limitations therein regarding generalizability and/or accuracy.

Option	Value	Definition
1	Yes	-
2	No	-
3	Can't tell	Assessment could not be made due to lacking reporting/data

G.5. Did the author(s) provide sufficient details and/or code to reproduce the steps described in this section?

Providing sufficient details and/or code[32] for the reader to understand and reproduce[33] the undertaken steps is essential in research in general[9,18] and in AI-based analyses specifically.[17] This enables critical appraisal of individual studies, tests of reproducibility, as well as meta-analysis or other forms of (quantitative) synthesis, ultimately accelerating advances in the field.[34]

Option	Value	Definition
1	Yes	-
2	No	-
3	Can't tell	Assessment could not be made due to lacking reporting/data

Section rating

Rating	Definition
Strong	- All items in the section are 1
Moderate	- G.1 and/or G.3 is 2 or 3, and the remaining items are 1
Weak	- G.2, G.4 or G.5 is 2 or 3 (regardless of the other items)

Overall rating

Rating	Definition
Strong	0 "weak" section ratings
Moderate	1 "weak" section rating
Weak	≥2 "weak" section ratings

References

1.Mamikutty R, Aly AS, Marhazlinda J. Selecting Risk of Bias Tools for Observational Studies for a Systematic Review of Anthropometric Measurements and Dental Caries among Children. *Int J Environ Res Public Health*. Aug 15 2021;18(16)doi:10.3390/ijerph18168623

2.Sanderson S, Tatt ID, Higgins JPT. Tools for assessing quality and susceptibility to bias in observational studies in epidemiology: a systematic review and annotated bibliography. *International Journal of Epidemiology*. 2007;36(3):666-676. doi:10.1093/ije/dym018

3.Kolaski K, Logan LR, Ioannidis JPA. Guidance to best tools and practices for systematic reviews. *Systematic Reviews*. 2023/06/08 2023;12(1):96. doi:10.1186/s13643-023-02255-9

4.Shea BJ, Reeves BC, Wells G, et al. AMSTAR 2: a critical appraisal tool for systematic reviews that include randomised or non-randomised studies of healthcare interventions, or both. *BMJ*. 2017;358:j4008. doi:10.1136/bmj.j4008

5.Whiting P, Savović J, Higgins JP, et al. ROBIS: A new tool to assess risk of bias in systematic reviews was developed. *J Clin Epidemiol*. Jan 2016;69:225-34. doi:10.1016/j.jclinepi.2015.06.005

6.Luo W, Phung D, Tran T, et al. Guidelines for Developing and Reporting Machine Learning Predictive Models in Biomedical Research: A Multidisciplinary View. *J Med Internet Res*. Dec 16 2016;18(12):e323. doi:10.2196/jmir.5870

7.Stevens LM, Mortazavi BJ, Deo RC, Curtis L, Kao DP. Recommendations for Reporting Machine Learning Analyses in Clinical Research. *Circulation: Cardiovascular Quality and Outcomes*. 2020/10/01 2020;13(10):e006556. doi:10.1161/CIRCOUTCOMES.120.006556

8.Shelmerdine SC, Arthurs OJ, Denniston A, Sebire NJ. Review of study reporting guidelines for clinical studies using artificial intelligence in healthcare. *BMJ Health Care Inform*. Aug 2021;28(1)doi:10.1136/bmjhci-2021-100385

9.van de Schoot R, Sijbrandij M, Winter SD, Depaoli S, Vermunt JK. The GRoLTS-Checklist: Guidelines for Reporting on Latent Trajectory Studies. *Structural Equation Modeling: A Multidisciplinary Journal*. 2017/05/04 2017;24(3):451-467. doi:10.1080/10705511.2016.1247646

10.Owora AH, Zhang Y. Childhood wheeze trajectory-specific risk factors: A systematic review and meta-analysis. *Pediatr Allergy Immunol*. Jan 2021;32(1):34-50. doi:10.1111/pai.13313

11.Higgins JPT, Altman DG, Sterne JAC, (editors). *Cochrane Handbook for Systematic Reviews of Interventions*. Version 5.2.0 (updated June 2017) ed.:chap 8: Assessing risk of bias in included studies. Accessed 28 August 2023. https://training.cochrane.org/sites/training.cochrane.org/files/public/uploads/resources/Handbook5_1/Chapter_8_Handbook_5_2_8.pdf

12.Armijo-Olivo S, Stiles CR, Hagen NA, Biondo PD, Cummings GG. Assessment of study quality for systematic reviews: a comparison of the Cochrane Collaboration Risk of Bias Tool and the Effective Public Health Practice Project Quality Assessment Tool: methodological research. *J Eval Clin Pract*. Feb 2012;18(1):12-8. doi:10.1111/j.1365-2753.2010.01516.x

13. Bashir MBA, Basna R, Zhang G-Q, et al. Computational phenotyping of obstructive airway diseases: protocol for a systematic review. *Systematic Reviews*. 2022/10/13 2022;11(1):216. doi:10.1186/s13643-022-02078-0
14. Meijs C, Handoko ML, Savarese G, et al. Discovering Distinct Phenotypical Clusters in Heart Failure Across the Ejection Fraction Spectrum: a Systematic Review. *Current Heart Failure Reports*. 2023/07/21 2023;doi:10.1007/s11897-023-00615-z
15. Stafford IS, Gosink MM, Mossotto E, Ennis S, Hauben M. A Systematic Review of Artificial Intelligence and Machine Learning Applications to Inflammatory Bowel Disease, with Practical Guidelines for Interpretation. *Inflamm Bowel Dis*. Oct 3 2022;28(10):1573-1583. doi:10.1093/ibd/izac115
16. Stafford IS, Kellermann M, Mossotto E, Beattie RM, MacArthur BD, Ennis S. A systematic review of the applications of artificial intelligence and machine learning in autoimmune diseases. *NPJ Digit Med*. 2020;3:30. doi:10.1038/s41746-020-0229-3
17. Allam A, Feuerriegel S, Rebhan M, Krauthammer M. Analyzing Patient Trajectories With Artificial Intelligence. *J Med Internet Res*. Dec 3 2021;23(12):e29812. doi:10.2196/29812
18. Kocak B, Kus EA, Kilickesmez O. How to read and review papers on machine learning and artificial intelligence in radiology: a survival guide to key methodological concepts. *European Radiology*. 2021/04/01 2021;31(4):1819-1830. doi:10.1007/s00330-020-07324-4
19. Faes L, Liu X, Wagner SK, et al. A Clinician's Guide to Artificial Intelligence: How to Critically Appraise Machine Learning Studies. *Transl Vis Sci Technol*. Feb 12 2020;9(2):7. doi:10.1167/tvst.9.2.7
20. Kukull WA, Ganguli M. Generalizability: the trees, the forest, and the low-hanging fruit. *Neurology*. Jun 5 2012;78(23):1886-91. doi:10.1212/WNL.0b013e318258f812
21. Althubaiti A. Information bias in health research: definition, pitfalls, and adjustment methods. *J Multidiscip Healthc*. 2016;9:211-7. doi:10.2147/jmdh.S104807
22. Leung L. Validity, reliability, and generalizability in qualitative research. *J Family Med Prim Care*. Jul-Sep 2015;4(3):324-7. doi:10.4103/2249-4863.161306
23. Bolarinwa OA. Principles and Methods of Validity and Reliability Testing of Questionnaires Used in Social and Health Science Researches. *Nigerian Postgraduate Medical Journal*. 2015;22(4)
24. Swanson E. Validity, reliability, and the questionable role of psychometrics in plastic surgery. *Plast Reconstr Surg Glob Open*. Jun 2014;2(6):e161. doi:10.1097/gox.0000000000000103
25. Scott PJ, Brown AW, Adedeji T, Wyatt JC, Georgiou A, Eisenstein EL, Friedman CP. A review of measurement practice in studies of clinical decision support systems 1998-2017. *J Am Med Inform Assoc*. Oct 1 2019;26(10):1120-1128. doi:10.1093/jamia/ocz035
26. Tavakol M, Dennick R. Making sense of Cronbach's alpha. *Int J Med Educ*. Jun 27 2011;2:53-55. doi:10.5116/ijme.4dfb.8dfd
27. Wu Y, Jia M, Xiang C, Lin S, Jiang Z, Fang Y. Predicting the long-term cognitive trajectories using machine learning approaches: A Chinese nationwide longitudinal database. *Psychiatry Research*. 2022/04/01/ 2022;310:114434. doi:https://doi.org/10.1016/j.psychres.2022.114434

28. Ibrahim JG, Molenberghs G. Missing data methods in longitudinal studies: a review. *Test (Madr)*. May 1 2009;18(1):1-43. doi:10.1007/s11749-009-0138-x
29. Desai RJ, Wang SV, Vaduganathan M, Evers T, Schneeweiss S. Comparison of Machine Learning Methods With Traditional Models for Use of Administrative Claims With Electronic Medical Records to Predict Heart Failure Outcomes. *JAMA Netw Open*. Jan 3 2020;3(1):e1918962. doi:10.1001/jamanetworkopen.2019.18962
30. Berisha V, Krantsevich C, Hahn PR, Hahn S, Dasarathy G, Turaga P, Liss J. Digital medicine and the curse of dimensionality. *NPJ Digit Med*. Oct 28 2021;4(1):153. doi:10.1038/s41746-021-00521-5
31. Mullin S, Zola J, Lee R, et al. Longitudinal K-means approaches to clustering and analyzing EHR opioid use trajectories for clinical subtypes. *Journal of Biomedical Informatics*. 2021/10/01/ 2021;122:103889. doi:https://doi.org/10.1016/j.jbi.2021.103889
32. Martin EH, Annie AB, Aidan GC, et al. Quality Output Checklist and Content Assessment (QuOCCA): a new tool for assessing research quality and reproducibility. *BMJ Open*. 2022;12(9):e060976. doi:10.1136/bmjopen-2022-060976
33. Plesser HE. Reproducibility vs. Replicability: A Brief History of a Confused Terminology. *Front Neuroinform*. 2017;11:76. doi:10.3389/fninf.2017.00076
34. Begley CG, Ioannidis JP. Reproducibility in science: improving the standard for basic and preclinical research. *Circ Res*. Jan 2 2015;116(1):116-26. doi:10.1161/circresaha.114.303819
35. Nguena Nguefack HL, Pagé MG, Katz J, et al. Trajectory Modelling Techniques Useful to Epidemiological Research: A Comparative Narrative Review of Approaches. *Clin Epidemiol*. 2020;12:1205-1222. doi:10.2147/clep.S265287
36. Kivimäki M, Singh-Manoux A, Ferrie JE, Batty GD. Post hoc decision-making in observational epidemiology--is there need for better research standards? *Int J Epidemiol*. Apr 2013;42(2):367-70. doi:10.1093/ije/dyt036
37. Li G, Taljaard M, Van den Heuvel ER, et al. An introduction to multiplicity issues in clinical trials: the what, why, when and how. *International Journal of Epidemiology*. 2017;46(2):746-755. doi:10.1093/ije/dyw320
38. Bender R, Lange S. Adjusting for multiple testing—when and how? *Journal of Clinical Epidemiology*. 2001/04/01/ 2001;54(4):343-349. doi:https://doi.org/10.1016/S0895-4356(00)00314-0
39. Efird JT, Nielsen SS. A method to compute multiplicity corrected confidence intervals for odds ratios and other relative effect estimates. *Int J Environ Res Public Health*. Dec 2008;5(5):394-8. doi:10.3390/ijerph5050394
