# Supplementary material for: Machine learning-derived phenotypic trajectories of asthma and allergy in children and adolescents: protocol for a systematic review
Source: BMJ Open. 2024 Aug 30;14(8):e080263. doi: 10.1136/bmjopen-2023-080263 (PMC11367367; doi:10.1136/bmjopen-2023-080263)
Supplement: Supplementary file 2 [file bmjopen-2023-080263.reviewer_comments.pdf]

PEER REVIEW HISTORY

BMJ Open publishes all reviews undertaken for accepted manuscripts. Reviewers are asked to complete a checklist review form and are provided with free text boxes to elaborate on their assessment. These free text comments are reproduced below.

ARTICLE DETAILS

**TITLE (PROVISIONAL)**	Machine learning-derived phenotypic trajectories of asthma and allergy in children and adolescents: protocol for a systematic review
**AUTHORS**	Lisik, Daniil; Milani, Gregorio; Salisu, Michael; Özuygur Ermis, Saliha Selin; Goksör, Emma; Basna, Rani; Wennergren, Göran; Kankaanranta, Hannu; Nwaru, Bright

VERSION 1 - REVIEW

**REVIEWER NAME**	Cooper, Philip
**REVIEWER AFFILIATION**	St George's University of London, Institute of Infection and Immunity
**REVIEWER CONFLICT OF INTEREST**	None.
**DATE REVIEW RETURNED**	09-Oct-2023

**GENERAL COMMENTS**	Overall, a well-described and designed systematic review addressing an area of scientific importance in our understanding of the development of allergic diseases and how they might be inter-related.<br><br>Presumably there is a knowledge gap here with no other similar systematic reviews currently registered?<br><br>Lines 78-80: “For example, recurrent wheezing during infancy is often the result of viral respiratory infections, while such symptoms during later childhood are more often caused by asthma”<br>A significant proportion of infants with recurrent wheezing are diagnosed with asthma in later childhood. Recurrent symptoms at both stages are primarily associated with respiratory viral infections although in the older group there may be an important interaction with allergic sensitization. Asthma does not cause recurrent wheezing, but rather recurrent wheezing is a manifestation of the disease and is caused by the pathophysiological process underlying this heterogeneous disease.<br><br>Eligibility criteria; Surely all studies should be longitudinal by definition if there are 2 or more prospective observations for each participant over a minimum of 1 year observation?<br><br>I can’t comment on the statistics but there may be some special statistical considerations to consider given the type of data being generated by these studies.<br><br>It might be useful to provide some text on potential limitations of the authors’ approach.

**REVIEWER NAME**	Tan, Daniel
**REVIEWER AFFILIATION**	University of Melbourne VCCC

**REVIEWER CONFLICT OF INTEREST**	No competing interests
**DATE REVIEW RETURNED**	14-Dec-2023

**GENERAL COMMENTS**	The authors have established a comprehensive and well written methodology for a systematic review in summarizing the current state of childhood machine learning-derived phenotypic trajectories of asthma and allergy. It will be helpful if possible to include types and frequency of early childhood infections together with types of diets the child may be exposed to and how that may affect the trajectory of allergy development.<br><br>Line 69, The author should provide a more definite number instead of hundreds or millions.

**REVIEWER NAME**	Lewis, Kate
**REVIEWER AFFILIATION**	UCL Great Ormond Street Institute of Child Health, Population Policy and Practice Research and Teaching Department
**REVIEWER CONFLICT OF INTEREST**	No competing interests to declare
**DATE REVIEW RETURNED**	27-Dec-2023

**GENERAL COMMENTS**	The proposed study is very relevant to the field of paediatric asthma and atopy, offering a comprehensive synthesis of the phenotyping literature. The authors acknowledge that there are existing systematic reviews on phenotypic trajectories, but intend to provide novel material by (1) "focusing on machine learning-derived phenotypic trajectories in children" and (2) by "encompassing a broad spectrum of allergic diseases as well as asthma". The methods are clearly described, and the PRISMA-P checklist included. However, the methods could be improved with a further clarification on what is meant by machine learning analyses in this context. It may be useful, for example, to list specific methods to include/exclude within the eligibility criteria to improve the sensitivity and specificity of the search.<br><br>Some minor comments:<br><br>- I wondered if you could add "date-driven" or similar into the title.<br><br>- P7 line 7-80 "For example, recurrent wheezing during infancy is often the result of viral respiratory infections, while such symptoms during later childhood are more often caused by asthma" - This could perhaps be rephrased as I'm not sure that the symptoms as caused by asthma, rather they are labelled as such.<br><br>- Reference 21 and 24 are duplicates.

**REVIEWER NAME**	Sousa, Tatiane
**REVIEWER AFFILIATION**	USP
**REVIEWER CONFLICT OF INTEREST**	There are no competing interests
**DATE REVIEW RETURNED**	23-Mar-2024

GENERAL COMMENTS	Dears,<br><br>I have reviewed the manuscript outlining the protocol for conducting a systematic review of the phenotypic trajectories of asthma and allergy in individuals under 18. The study's objectives to summarize the identified trajectories, along with the variables and machine learning approach utilized, are commendable and contribute significantly to the field.<br><br>Furthermore, the methodological steps proposed are detailed, and the supplementary material provides valuable insights into the protocol to be adopted. Throughout the review process, I have identified only minor revision suggestions, which will enhance the manuscript's clarity and comprehensiveness.<br><br>Therefore, I recommend it for publication and am grateful for the opportunity to have been involved in its review process.<br><br>My recommendations are provided below, organized according to the manuscript sections.<br><br>Abstract<br><br>Line 34 - The authors mention that the study pertains to "asthma and allergies in children," but at other times, it seems the study also includes adolescents. I suggest explicitly stating at the beginning which population group is of interest.<br><br>I suggest incorporating the "trajectories studies" approach in the Introduction to define it and clarify the authors' approach.<br><br>Strengths and Limitations<br><br>The authors mention "computational trajectory exploration" in the abstract and "machine learning-derived phenotypic trajectories" in this section. I suggest mentioning machine learning in the abstract.<br><br>Introduction<br><br>Line 97 - The authors mention that the study includes the population under 18 years old. This would no longer be only children but also adolescents. I suggest clarifying and standardizing the target audience throughout the text and the title.<br><br>Line 99 - Specify which trajectories are referred to.<br><br>Methods<br><br>Line 163/164 - I suggest including the date of the pilot study in PubMed.<br><br>Line 383 - I suggest that the authors add more details of the statistical analysis, mainly related to the "CORR" or "HIER" model weighting scheme. For example, the authors say that "the default rho value of 0.8 will be used" but do not provide a reference or explanation.<br><br>Line 420 - I suggest that the authors include a justification for using the Begg and Mazumdar correlation and Egger's regression tests.

	In the statistical analyses, it is unclear if the sample size of the studies obtained will be considered in the meta-analysis.<br><br>The authors mention that the systematic review is dedicated to studies on children, but it refers to the population under 18 years old and includes words like "teenagers" in the search strategies, as presented in the supplementary material. Therefore, I suggest revising the title and the manuscript to include a reference to the entire studied population (children and adolescents). Furthermore, I suggest the authors consider the age groups in their analyses since asthma and allergy trajectories may differ between children and adolescents.<br><br>Supplementary Material<br><br>It is not possible to read the information included in the Full query.<br><br>Table 7 - I suggest using a traditional format to present the trajectory characteristics and associations. The proposed format is not so intuitive to understand the results, including the information presented in the colored boxes.

VERSION 1 – AUTHOR RESPONSE

Reviewer 1

Presumably there is a knowledge gap here with no other similar systematic reviews currently registered?

That is correct. Prior to initiating the project, we performed a review of the published literature to see if there are any published systematic reviews with identical or similar aims as the present work. This was done as not all systematic reviews register their work on protocol platforms. In addition, we searched for related keywords on PROSPERO to assess if anyone planned similar work. In summary, we did not identify any planned or published systematic review which would render the present work superfluous. We shortly described this on page 4, lines 109-116.

Lines 78-80: “For example, recurrent wheezing during infancy is often the result of viral respiratory infections, while such symptoms during later childhood are more often caused by asthma”

A significant proportion of infants with recurrent wheezing are diagnosed with asthma in later childhood. Recurrent symptoms at both stages are primarily associated with respiratory viral infections although in the older group there may be an important interaction with allergic sensitization. Asthma does not cause recurrent wheezing, but rather recurrent wheezing is a manifestation of the disease and is caused by the pathophysiological process underlying this heterogeneous disease.

Thank you for pointing out this important aspect, which is true. Our rationale with the referenced text was to highlight that certain clinical presentations/symptoms may reflect differing underlying conditions depending on age. However, you are indeed correct that a large proportion of infants with recurrent wheezing end up developing (or are found out to have from an early age) proper asthma. For this reason, we removed this section, and replaced it with a short example of how risk factors may differ in association with different trajectories of atopic disease (page 4, lines 96-99), to provide a reasoning for the inclusion of risk factor meta-analysis in our work.

Eligibility criteria; Surely all studies should be longitudinal by definition if there are 2 or more prospective observations for each participant over a minimum of 1 year observation?

Our thinking was that there may potentially be studies out there in which subjects were asked about symptoms or the presence/absence of disease at two different time points 12 months or more apart, but indeed you are right that realistically, the relevant studies are all longitudinal. We have thus redefined this statement (page 5, line 150).

I can't comment on the statistics but there may be some special statistical considerations to consider given the type of data being generated by these studies.

Indeed the data investigated encompass multiple types and necessitate considerations regarding suitable statistical approaches. We have written about what we believe are the most essential aspects, e.g., the differential meta-analysis approach depending on data type (robust variance estimation for risk factors and outcomes, given their commonly non-independent nature [e.g., investigation of birth order 2 vs 1 and ≥3 vs 1 as risk factor for a trajectory, and thus re-utilizing the same reference group twice, thus inducing bias in a traditional meta-analysis model; pages 13-14, lines 414-423] and generalized linear mixed models for static subject characteristics [pages 15, lines 453-457]). Additional considerations discussed include the assessment of hetereogeneity in the data through subgroup analysis (page 13, lines 406-407), sensitivity analysis (page 15, lines 463-464), as well as evaluation of statistical significance (page 14, lines 440-443).

It might be useful to provide some text on potential limitations of the authors' approach.

Thank you for bringing this point up. We have now added this to the Discussion (page 16, lines 489-499).

Reviewer 2

The authors have established a comprehensive and well written methodology for a systematic review in summarizing the current state of childhood machine learning-derived phenotypic trajectories of asthma and allergy. It will be helpful if possible to include types and frequency of early childhood infections together with types of diets the child may be exposed to and how that may affect the trajectory of allergy development.

Thank you for these inputs and suggestions. We are indeed including these factors as well in the association analyses. It has been added to the list on page 13, lines 393-394.

Line 69, The author should provide a more definite number instead of hundreds or millions.

Thank you for this comment. Although "hundreds of millions" can be said of highly different absolute numbers, we chose this term because it reflects the magnitude while not being overly specific, which would introduce other issues, as regional differences in prevalence are substantial. Likewise, prevalence differs by age as well as specific disease. Thus, to make for smooth reading while conveying the message that these conditions are common in childhood, we chose this formulation. To further emphasize the commonness of asthma and allergy in childhood, we added a few words on the prevalence in the first sentence of the Introduction (page 4, line 87).

Reviewer 3

The proposed study is very relevant to the field of paediatric asthma and atopy, offering a comprehensive synthesis of the phenotyping literature. The authors acknowledge that there are existing systematic reviews on phenotypic trajectories, but intend to provide novel material by (1) "focusing on machine learning-derived phenotypic trajectories in children" and (2) by "encompassing a broad spectrum of allergic diseases as well as asthma". The methods are clearly described, and the PRISMA-P checklist included. However, the methods could be improved with a further clarification on what is meant by machine learning analyses in this context. It may be useful, for example, to list

specific methods to include/exclude within the eligibility criteria to improve the sensitivity and specificity of the search.

Thank you for these words! Our aim is to be very inclusive in terms of machine learning techniques accepted for inclusion, as this is very much an aspect of interest for us, namely to evaluate the latest trends in computational methodology, including the very choice of trajectory analysis algorithm. This has also been clarified in the initial version on pages 6, lines 162-166).

Some minor comments:

- I wondered if you could add “date-driven” or similar into the title.

Thank you for this suggestion! We fully agree that it would be a good idea to emphasize this aspect in the title and have now added "Machine learning-based" to the title, to clearly indicate that the trajectories investigated will be those identified with such techniques.

- P7 line 7-80 “For example, recurrent wheezing during infancy is often the result of viral respiratory infections, while such symptoms during later childhood are more often caused by asthma” - This could perhaps be rephrased as I’m not sure that the symptoms as caused by asthma, rather they are labelled as such.

Thank you for pointing this out. You are correct indeed, and as another reviewer also pointed out, both recurrent wheezing in infancy and later childhood alike oftentimes develop (or manifests) proper asthma. This section has now been replaced (page 4, lines 96-99) by an example of how risk factors are differentially associated with different asthma/allergy trajectories, indicating the importance of characterizing associated factors and not only the longitudinal disease patterns alone.

- Reference 21 and 24 are duplicates.

Thank you for taking notice of this. We have now exchanged the second duplicate citation with the first Cunha et al. reference (page 4, lines 105 and 111).

Reviewer 4

Abstract

Line 34 - The authors mention that the study pertains to "asthma and allergies in children," but at other times, it seems the study also includes adolescents. I suggest explicitly stating at the beginning which population group is of interest.

Thank you for the kind words in the preceding sections of your comment, and for this suggestion. We agree that the text would benefit from clarification on this, as adolescence in particular is a very special age, bridging childhood and adulthood. We have now added this to the Abstract (page 2, lines 35 and 39-40).

I suggest incorporating the "trajectories studies" approach in the Introduction to define it and clarify the authors' approach.

Good idea, thank you! We have now added this to the Introduction in the Abstract (page 2, line 39).

Strengths and Limitations

The authors mention "computational trajectory exploration" in the abstract and "machine learning-derived phenotypic trajectories" in this section. I suggest mentioning machine learning in the abstract.

We agree that a more consistent terminology is beneficial. We have rephrased the Abstract sentence in question (page 2, line 39).

Introduction

Line 97 - The authors mention that the study includes the population under 18 years old. This would no longer be only children but also adolescents. I suggest clarifying and standardizing the target audience throughout the text and the title.

You are very right that referring to individuals reaching the age of 18 years as adolescents is more appropriate. We have now edited the title and other instances where the word "children" occurs to also encompass "adolescents" (page 4, lines 89 and 114; page 5, lines 130 and 153).

Line 99 - Specify which trajectories are referred to.

We have now specified this (page 5, line 130).

Methods

Line 163/164 - I suggest including the date of the pilot study in PubMed.

Thank you for this suggestion. We do not have one specific date during which the piloting of the search queries was performed, as this was done over the course of multple days in September 2023. We have now added this information (page 7, line 197).

Line 383 - I suggest that the authors add more details of the statistical analysis, mainly related to the "CORR" or "HIER" model weighting scheme. For example, the authors say that "the default rho value of 0.8 will be used" but do not provide a reference or explanation.

We agree fully that these terms should be explained in more detail. We have now expanded the section on "CORR" and "HEIR" to describe their meaning a bit more (page 14, lines 425-429). In regards to the rho value, the default value has been shown to be well-suited for most contexts. For this reason, in many well-designed meta-analyses, this default value is used without even running a sensitivity analysis (e.g., this review by Zhang et al: https://doi.org/10.1371/journal.pmed.1003731). In a previous related work, we performed sensitivity analysis (i.e., re-running the meta-analysis with rho values ranging from the minimum allowed (0) to the maximum allowed (1) value, finding no meaningful difference. This work has now been referenced (page 14, line 431).

Line 420 - I suggest that the authors include a justification for using the Begg and Mazumdar correlation and Egger’s regression tests.

We utilized the abovementioned tests for two reasons: (1) they are commonly utilized and versatile; (2) it has been recommended by many experts that relying on just one measure is insufficient. We have now added a reference of an empirical comparison study to strenghten these justifications (page 15, line 466 [right before the colon]).

In the statistical analyses, it is unclear if the sample size of the studies obtained will be considered in the meta-analysis.

We thank the reviewer for this comment and have taken it into consideration, but we do not plan to directly perform any subanalyses based on sample size, as we find it a weaker indicator of estimate/trajectory validity than e.g., overall methodological quality (proxied by the overall rating), which we will perform sensitivity analysis on.

The authors mention that the systematic review is dedicated to studies on children, but it refers to the population under 18 years old and includes words like "teenagers" in the search strategies, as presented in the supplementary material. Therefore, I suggest revising the title and the manuscript to include a reference to the entire studied population (children and adolescents). Furthermore, I

suggest the authors consider the age groups in their analyses since asthma and allergy trajectories may differ between children and adolescents.

You are correct that "adolescence" is a more suitable description to individuals in-between what we commonly refer to as childhood and adulthood. We have now added "adolescence" to the subject descriptions where "childhood" or "children" is mentioned (please see above points). In regards to the last sentence in this comment, we agree fully. We have now added this aspect to specifically assess when pooling estimates (page 13, line 404). In addition, the age during which the trajectory-defining data were collected also constitues an important aspect for combining trajectories descriptively, as e.g., "late-onset" can be very different depending on the time frame during which subjects were followed. We have now added this as well (page 11, lines 353-354).

Supplementary Material

It is not possible to read the information included in the Full query.

We agree that it would be a good idea to make this text block at least readable. We have thus increased the size of the text blocks in question from 2 to 6.5 points (Supplementary tables 2A-J).

Table 7 - I suggest using a traditional format to present the trajectory characteristics and associations. The proposed format is not so intuitive to understand the results, including the information presented in the colored boxes.

Thank you for this comment and input. We agree that the table structure proposed could be simplified and made more intuitive. We have thus heavily revised the structure of the table in question in order to make it less cluttered but keep practically all information as before (Supplementary table 7). Regarding the colored boxes, we understand that the coloring is not universally recognized, and will likely not aid the reader much in navigating the tables. We have thus skipped the coloring aspect. However, we kept the table in question (Supplementary table 8), as we find it to be a reasonable approach to showcase the different variables that we will assess in this work in a structured manner. We hope that this is a reasonable compromise.

We hope that the revised manuscript is to your satisfication and acceptable for BMJ Open.

Sincerely,

Daniil Lisik, on behalf of all authors

VERSION 2 – REVIEW

**REVIEWER NAME**	Sousa, Tatiane
**REVIEWER AFFILIATION**	USP
**REVIEWER CONFLICT OF INTEREST**	There are no competing interests.
**DATE REVIEW RETURNED**	30-Jul-2024

**GENERAL COMMENTS**	The manuscript has been thoroughly revised by the authors, considering all the revisions provided. I greatly appreciate the consideration and responses to the comments. The authors have chosen an important topic and adopted an appropriate and rigorous methodological approach.

VERSION 2 – AUTHOR RESPONSE

Reviewer 4

The manuscript has been thoroughly revised by the authors, considering all the revisions provided. I greatly appreciate the consideration and responses to the comments. The authors have chosen an important topic and adopted an appropriate and rigorous methodological approach..

Thank you for the kind and encouraging words and for dedicating time to review this work!
